# Towards ultra-fast treatments: large energy acceptance beam delivery systems and opportunities for proton beam therapy

**DOI:** 10.3389/fonc.2026.1791102

**Published:** 2026-06-03

**Authors:** Jacinta Yap, Adam Steinberg, Hannah Norman, Konrad P. Nesteruk, Suzie Sheehy

**Affiliations:** 1 Department of Radiation Oncology, Peter MacCallum Cancer Centre, Melbourne, VIC, Australia; 2 School of Physics, University of Melbourne, Melbourne, VIC, Australia; 3 Cockcroft Institute, Daresbury, Warrington, United Kingdom; 4 Department of Physics and Astronomy, University of Manchester, Manchester, United Kingdom; 5 Department of Radiation Oncology, Mass General Brigham Cancer Institute and Harvard Medical School, Boston, MA, United States; 6 Australian Nuclear Science and Technology Organisation (ANSTO), Lucas Heights, NSW, Australia

**Keywords:** beam delivery, compact facilities, large energy acceptance, novel delivery modalities, particle therapy, proton beam therapy, rapid delivery

## Abstract

The availability of proton beam therapy (PBT) continues to grow exponentially worldwide, driven by technological advancements to reduce the facility size and costs, towards more efficient and higher quality treatments. The characteristic physical and biological advantages of protons can provide superior clinical outcomes for patients, as modern techniques enable a highly configurable and conformal dose delivery. Although active scanning methods allow precise beam control, proton beams are highly sensitive to range and motion errors which impact treatment quality. Treatment delivery is largely determined by capabilities of the beam delivery system (BDS), where faster delivery can have many potential benefits including improved dosimetric quality, utility, cost effectiveness, patient throughput and comfort. Despite significant developments in accelerators, delivery methodologies, dose optimisation and more, the energy layer switching time (ELST) is still a persisting limitation in existing beam delivery systems. The ELST can be a major contributor to the irradiation time, leading to increased treatment times, and may require further compensation using optimisation planning approaches, motion mitigation strategies, or active beam modification. This fundamental constraint can be addressed by increasing the narrow energy acceptance range of conventional beamlines to allow a wide range of beam energies to be transported without bottleneck delays due to magnetic field adjustments, therefore minimising ELSTs and enabling ultra-fast delivery (single field within ∼10 s). We review the abundant opportunities offered by this enabling technology: shorter treatment times, reduced motion induced dose degradation, improved effectiveness of motion management techniques, possibilities for volumetric rescanning, bidirectional delivery, novel planning optimisation schemes, and emerging delivery strategies. We overview the design concepts of several large energy acceptance (LEA) proposals, technology requirements, and also discuss the remaining challenges and considerations with realising a LEA system in practice. Although there are multiple avenues requiring further development and study, a large energy acceptance BDS has the potential for significant clinical benefits: ultra-fast delivery offers both immediate improvements to current treatment delivery and enables future possibilities for PBT.

## 1 Introduction

Proton beam therapy is a well established modality of radiotherapy cancer treatment and one of the most advanced and precise techniques available. Using a beam of protons can be clinically advantageous compared to conventional X-ray radiotherapy as the characteristic ‘Bragg Peak’ (BP) enables radiation to be delivered to targeted sites with greater accuracy and radiobiological impact (1). Modern PBT delivery – pencil beam scanning (PBS) – utilises the high energy deposition at the BP to achieve an extremely precise and conformal dose distribution throughout the entire tumour volume, sparing healthy surrounding tissue. This makes PBT highly suited for deep-seated cancers and tumours situated near critical organs. The dose sparing of normal tissue lowers the risk of damaging side effects, improving tolerance to treatment and a better quality of life for patients. New possibilities with multi-modality or combined therapies (i.e. immunotherapy) could also lead to better clinical outcomes (2).

**Figure 1:**
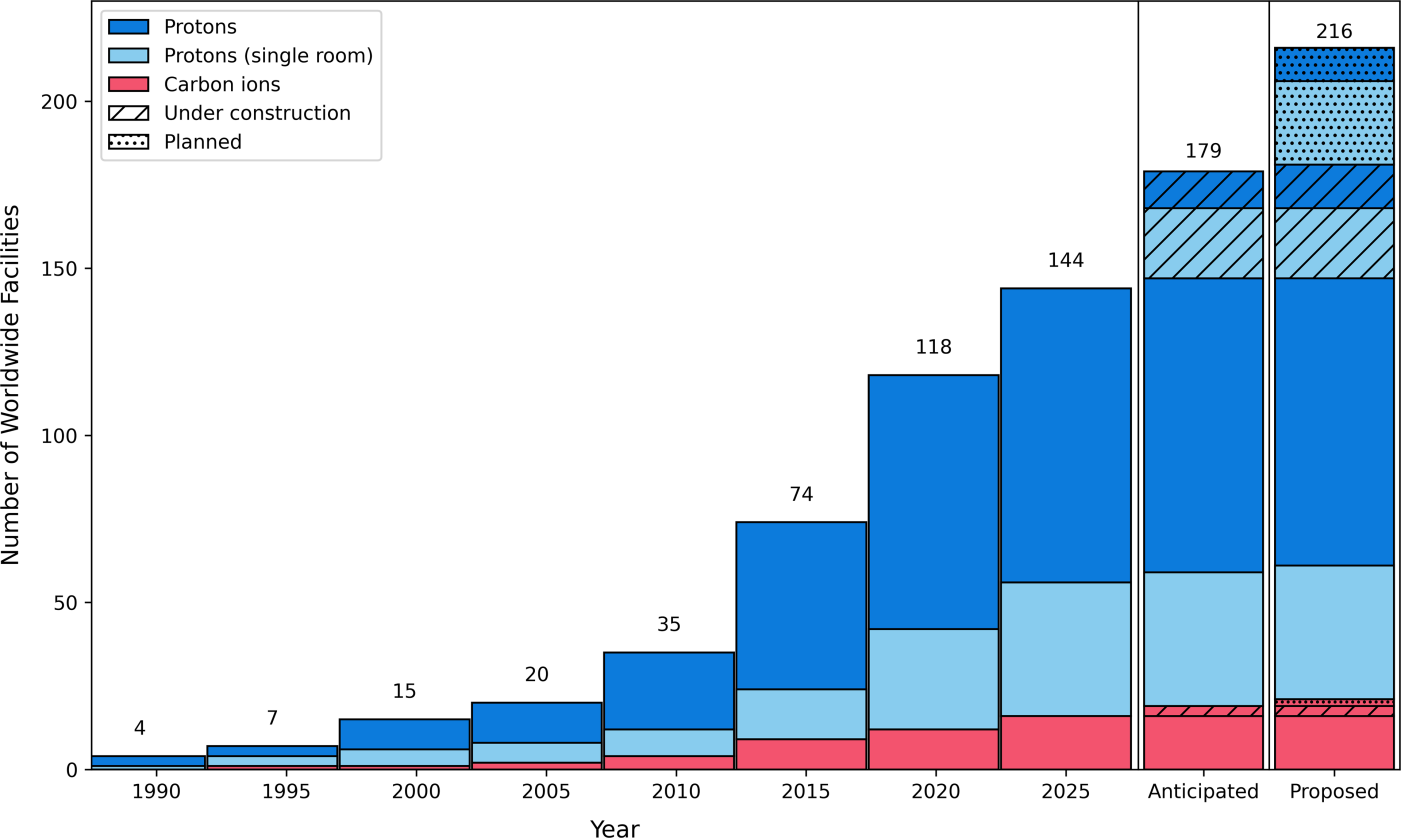
Worldwide PBT and CIBT facilities: operational, anticipated (under construction) and proposed (planned), total number of facilities are listed above bars (those listed to offer both PBT and CIBT are counted separately to reflect total availability). Single room PBT facilities are those reported with one beam or gantry – including dedicated ocular and upright centres. PTCOG data updated Feb 2026 (6).

Radiotherapy is involved in the treatment of approximately 40% of all cancer patients (3, 4): up to 50% of these patients could benefit from PBT yet <1% currently receive it (5). Presently there are >140 operating facilities which offer either proton or carbon ion beam therapy (CIBT) i.e. 128 PBT and 16 CIBT, Jan 2026 (6)). Although PBT availability is increasing worldwide (**Figure 1**), the global burden of cancer is growing: 50-70% of cancer patients need radiotherapy (7) yet many barriers still prohibit widespread adoption. Although considerable advances in accelerator and delivery technologies – particularly associated with the size and complexity of machines^1^ – have made it easier and more affordable to provide PBT, a significant and prevailing hurdle to access is the high cost (8).

**Figure 2:**
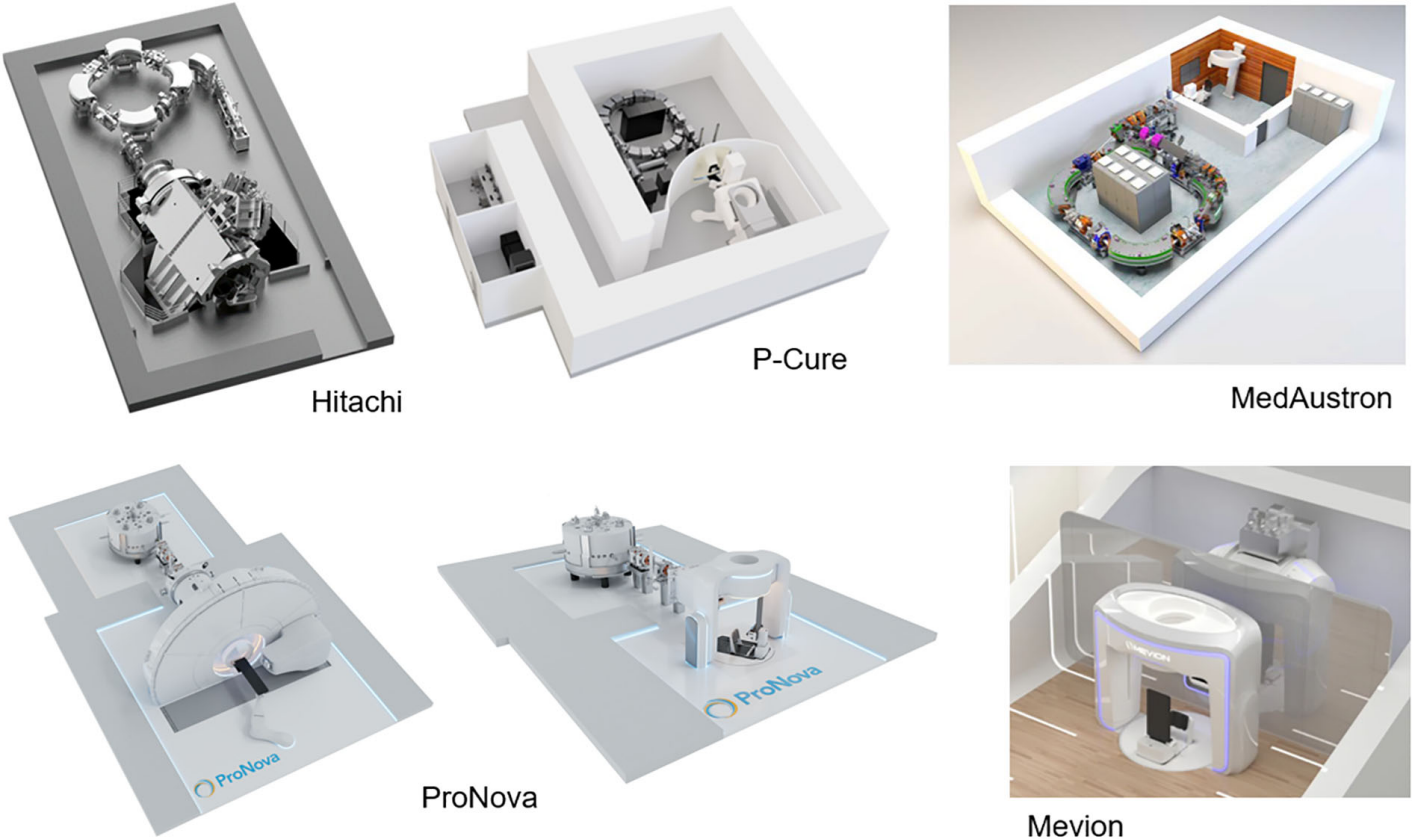
Compact, single-room PBT systems with gantries (left) and upright systems (middle, right). Hitachi image - reproduced from citation (10), CC-BY-4.0. P-Cure and MedAustron synchrotron solutions (top), ProNova and Mevion upright solutions with the Leo Cancer Care Marie chair (bottom, right). Images reproduced with individual permissions provided by P-cure, MedAustron, ProNova Solutions and Mevion Medical Systems images.

Recent growing interest in reduced footprints and costs have led to significant investment in smaller accelerators and therefore ‘single-room’ systems: ∼65% of facilities either under construction or planned are listed as having only one treatment room (6). The majority of upcoming PBT facilities are single-room systems with superconducting synchrocyclotrons such as the IBA ProteusOne (9), gantry-less Mevion S250-FIT or ProNova SC360. Compact synchrotrons are now available from a range of vendors and have been installed at several sites by Hitachi (PROBEAT) (10), P-Cure (11) and Protom (Radiance 330) (12). An alternate design (Compact 200+) has also been proposed by MedAustron with upright, couch or gantry configurations (**Figure 2**). A facility which can accommodate both the accelerator and treatment room within a single room (or similarly sized conventional linac bunkers) can be ∼USD $30-50M (8, 13) compared to ∼USD $100-200M (14, 15) for a multi-room facility – the standard route in previous decades. There is developing ambition for compact technologies, given that the BDS^2^ is a major contributor to the capital costs and physical space requirements. Most BDS include a gantry, a massive mechanical structure supporting a series of magnets to steer the beam to the patient. A gantry-less solution enables the possibility of retrofitting into existing facilities (16, 17), also encouraging greater worldwide PBT adoption (5).

The future technological requirements to fully exploit PBT are well recognised (18): there is a clear evolution of the field striving for better treatment efficiency (cost reduction, improved patient throughput and caseload utility) and efficacy (treatment quality). Much of the R&D progress made in recent years has resulted in improvements to accelerators, beam transport, delivery methodologies, dose optimisation methods and many more (19–26). This has led to cost reduction in providing PBT and continues to be the trend today (2, 27, 28). Despite progress, even if cost-effective treatments and universal patient access to PBT are realised, patients will be unable to completely benefit from the dosimetric and therapeutic advantages offered by PBT unless persistent limitations in current technologies are overcome.

A key constraint in the beam delivery process is the time delay incurred in varying the magnetic fields and beamline components: the energy layer switching time. One approach which could address this is to increase the momentum acceptance range by design (i.e. a large momentum acceptance or large energy acceptance BDS) to allow a wider range of beam energies to be transported and delivered to the patient, without changing any magnetic settings. In principle, accepting a larger beam momentum range bypasses restrictions imposed by existing BDS designs and eliminates the ELST bottleneck, speeding up energy changes for longitudinal layer coverage. This could result in a reduction of ELSTs by orders of magnitude down to 10’s ms, enabling significantly faster beam delivery and the capability of enhanced delivery methodologies (volumetric rescanning, FLASH and arc therapies etc.) which are challenged by technical limitations and require substantially faster delivery efficiency. The potential improvement to PBT treatments could be revolutionary: a LEA system enables immediate clinical benefits with ultra-fast delivery and the possibility to deliver advanced techniques and novel modalities.

One crucial aspect which forms the focus of this review article is the delivery speed, including the role of the BDS and restrictions with treatment efficiency. Faster beam delivery brings potential clinical and economic benefits, in addition to an improved patient experience. We explore opportunities to improve the beam delivery process by increasing beamline momentum acceptance and minimising the ELST, enhancing scanning capabilities. We examine the clinical advantages of a LEA BDS enabling ultra-fast delivery, given the direct benefits to treatment times, motion management, optimised delivery, and new possibilities. Increasing the momentum acceptance range has been a design objective explored in many conceptual proposals, but a LEA beam delivery system has yet to reach clinical implementation. An overview of existing LEA proposals are presented, comparing their design features, optics, magnet technology and feasibility. Finally, we identify key considerations for beam quality and translation of this technology into clinical practice, examining approaches for realistic performance and operation.

## 2 The case for faster beam delivery: opportunities and challenges

Beam delivery for PBT is a complex process and many interconnected factors determine how quickly treatment can be delivered. The energy layer switching time is a significant contributor to the BDT and reducing this bottleneck can offer many potential benefits for treatment. In this section we briefly overview treatment delivery and discuss several different areas which impact treatment delivery efficiency: existing technology and aspects which determine ELST (beamline acceptance range, accelerator and modulation schemes), motion mitigation approaches, and delivery methodologies (optimisation and novel approaches). We discuss the current status and challenges imposed by technological limitations, and the opportunities for ultra-fast delivery made possible with minimised ELSTs and a large energy acceptance.

Most modern PBT facilities have adopted technologies to deliver treatments using PBS. A highly conformal and optimised dose distribution can be achieved by changing the beam intensity, energy, and transverse position e.g. intensity modulated proton therapy (IMPT). The beam can also be delivered from multiple angles, typically with gantries which rotate to direct the beam to the patient. The main methods of spatially controlling the beam is by spot, line, or raster scanning, however the entire delivery process itself is multifaceted: there are many different technological components and systems (accelerator, beam transport, and control systems etc.) responsible for beam production, transport, and delivery. The beam delivery time (BDT) – which includes the duration of active irradiation and beam preparation – can vary significantly depending on the target volume, characteristics of the BDS, accelerator output, transmission capabilities and various other technical parameters as described in (18).

**Figure 3:**
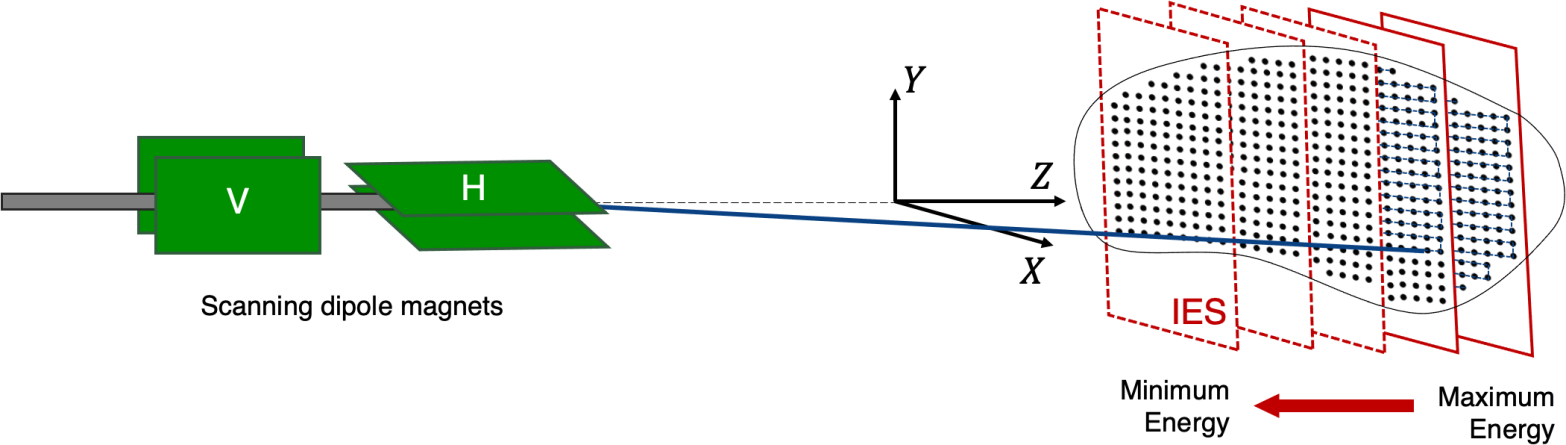
Schematic showing typical PBS delivery. 3D coverage of the treatment volume is achieved by scanning the beam across a layer (in the X-Y plane) in a predetermined pattern before the energy is lowered, reaching a shorter depth (in Z), then scanned across and repeated again for each consecutive layer. Adapted from citation (18), CC-BY-4.0

In PBS, a narrow beam scans across a transverse layer or ‘iso-energy slice’ (IES) in a pre-programmed pattern before the energy of the particle beam is reduced and the beam is scanned again. This is repeated for successive layers until there is complete coverage of the whole volume (**Figure 3**). This 3-dimensional distribution of dose reduces excess radiation outside the target site, minimising damage to healthy tissue and the risk of side effects.

**Figure 4:**
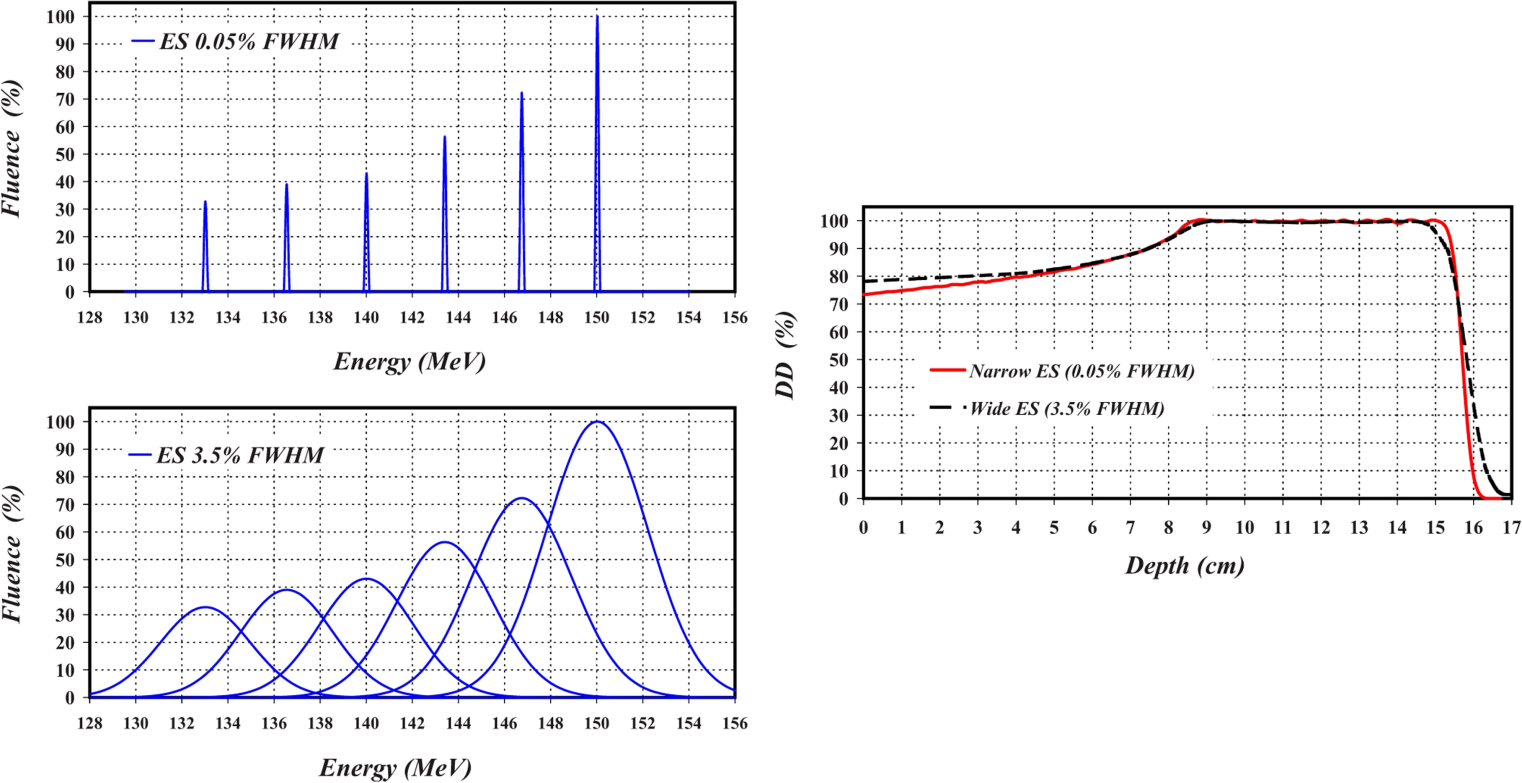
Energy modulation schemes depicting beams with a narrow (0.05%, top left) and large (3.5%, bottom left) incident energy spread (ES) and their resulting spread out BPs (right). A small beam energy spread may result in lower surface dose and a sharper distal fall-off however is less robust to range uncertainties and requires more layers for full coverage. Reproduced from citation (35), permissions granted by Rightslink (John Wiley and Sons).

The resulting dose distribution is highly configurable: delivery parameters can in principle be varied according to the treatment objectives and plan, including the scanning path or pattern, number of fields or spots, amount of time the beam dwells at an individual position (or it may be irradiating whilst moving along a path, such as in continuous scanning (29)), size of the beam spot, longitudinal beam spread, and spacing between the IES and spots. Many of these can be varied with the treatment planning software (TPS), using different optimisation algorithms or strategies (i.e. single field vs. multi-field optimisation) to provide the best quality treatment given a balance of computational demand, robustness and treatment time (30). For example, smaller spot sizes can achieve greater conformity (31, 32) but larger spots have higher plan robustness (33), and larger spot spacing can reduce treatment time but will degrade the homogeneity (34). Many narrow BPs may result in a smaller entrance plateau and steep distal SOBP fall-off but requires many closely spaced IES for a uniform distribution (35) (**Figure 4**). In contrast, a wider BP increases the IES spacing, requiring less layers for coverage but may reduce conformity (36). Further to this, fundamental issues such as patient and tumour motion will also have an inevitable impact on treatment quality (37, 38) and beams with a sharp energy distribution may be more susceptible to machine uncertainties. Therefore there are many parameters requiring consideration with planning and optimisation, more is discussed later in Section 2.4.

Consequently, as the delivery of hundreds of thousands of narrow beams are required for coverage of the entire tumour volume, PBS is inherently slower than passive scattering. Changing the depth of the BP requires complex adjustments – with the machine, magnets and control systems – and the time taken can be particularly lengthy relative to the total BDT: this energy layer switching time is a recognised, underlying bottleneck (18). Especially for large or complex cases which require many energy layers, and facilities with machines with longer ELSTs, the accumulation of time spent unnecessarily waiting becomes increasingly significant. Studies performed at clinics to model delivery parameter timings estimate the ELST can contribute up to 70-90% to the total BDT (independent of tumour characteristics) (39, 40).

Efficiency gains in the BDT achieved through better system and software optimisation to reduce the ELST, and facility scheduling utilisation have also demonstrated substantial improvements in uptime, clinical capacity, and workflow. A facility which implemented these upgrades was able to shorten their BDTs by more than a factor of 3, resulting in more fields and fractions being delivered and therefore higher patient throughput even with a mixed case load (41). The authors of this study also remark that faster treatments have the added benefit of allowing clinical staff more time to engage patients within the treatment time slot and less time may be spent immobilised in discomfort: important improvements to the overall patient experience.

### 2.1 Energy layer switching time

The energy layer switching time is a limiting factor in delivery efficiency, ranging anywhere on the order of ∼100’s ms up to a few seconds, depending on the accelerator and method of energy variation. An overview of reported ELSTs by several facilities given the main PBT accelerator types are listed in **Table 1**.

**Table 1:** Overview of reported ELSTs by accelerator type (vendor and model, if any) and facility.

ELSTs	Accelerator	Facility	Reference
80 ms (Gantry 2) 200 ms (Gantry 3)	Cyclotron	PSI, Switzerland	(42)
800 ms	Cyclotron (Varian ProBeam)	Emory Proton Therapy Center, USA	(43)
900 ms	Cyclotron (IBA C230)	Willis-Knighton Cancer Center, USA	(9)
500 ms	Cyclotron (IBA ProteusPLUS^®^ 235)	ProCure Proton Therapy Center, USA	(41)
220 ms (MEE)	Synchrotron	HIMAC, Japan	(44)
2.1 s	Synchrotron (Hitachi PROBEAT)	MD Anderson, USA	(39)
1.91 s 200 ms (MEE)	Synchrotron (Hitachi PROBEATV)	Mayo Clinic, USA	(40, 45)
2 s	Synchrotron	MedAustron, Austria	(46)
700 ms	Synchrocyclotron (IBA ProteusONE^®^ S2C2)	Beaumont Proton Therapy Center, USA	(47)
50 ms	Synchrocyclotron (Mevion S250i)	MAASTRO Proton Therapy, The Netherlands	(48)

ELSTs achieved using multiple energy extraction (MEE) are indicated.

For a synchrotron based facility the ELST is on the order of seconds, as it is dependent on the ramping speeds of the main ring magnets, extraction mechanism, or other features which are described in (49). This includes the time it takes to inject, accelerate, extract, decelerate and/or dump the remaining particles in each spill, for a single energy layer. To speed up IES changes, hybrid methods have been developed implementing a downstream range modulating device coupled with enhanced extraction operation (multiple energy, flattop extraction), allowing different energies to be produced within a single spill (50, 51). The ability to modulate energies with the accelerator itself provides the advantage of not needing a degradation system which requires activation, radiation protection and transmission loss considerations. For heavier ions which have a narrower BP, the accumulation of layers can result in a fluctuation of dose at the top of the SOBP distribution and beam modulation devices such as ripple filters (52) are used to broaden BPs and produce a smoother SOBP plateau as well as also decrease the number of IES, therefore speeding up the BDT. For PBT, ripple filters (or ridge filters (53)) could also be used to reduce the number of layers and dose gradient, thus also decreasing the sensitivity to machine range errors and the interplay effect (54).

**Figure 5:**
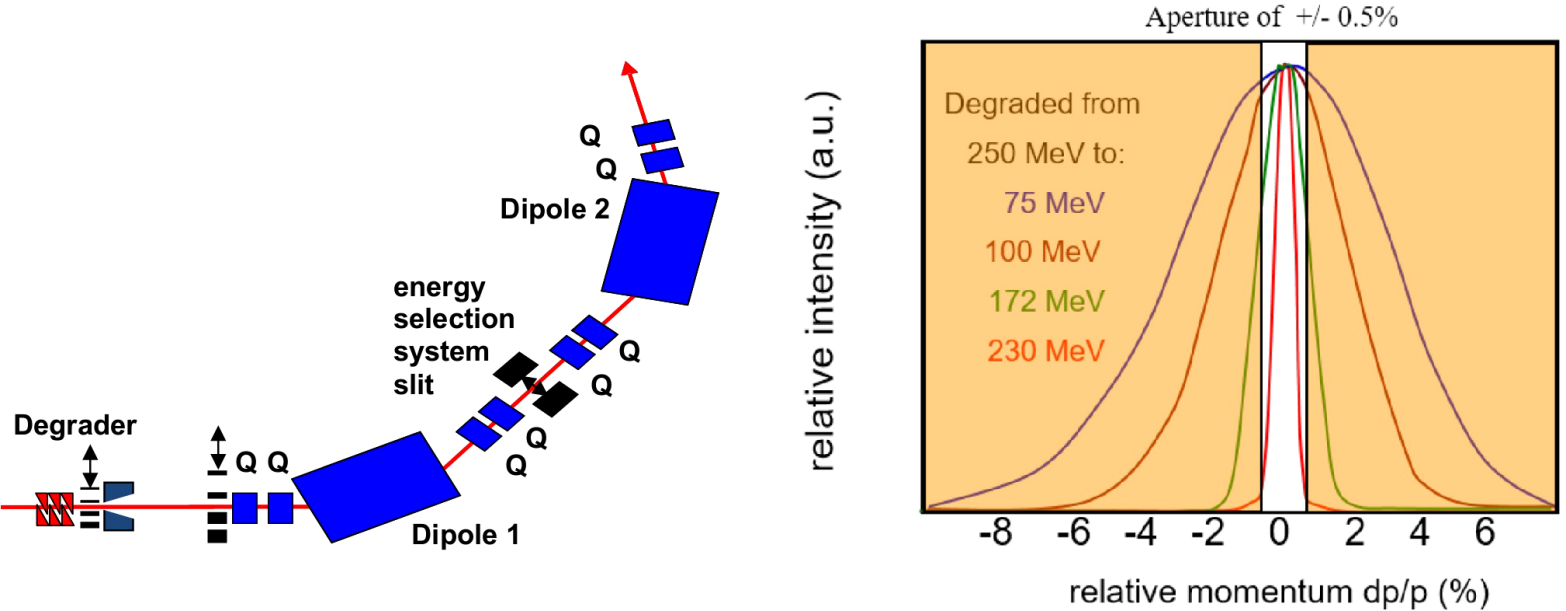
Schematic showing components which may typically comprise an ESS (left), reproduced from citations (159), CC-BY-4.0. Beam energies are reduced after passing through the degrader, resulting in particles with a spread of momenta (right). The ESS aperture indicates the distribution of particles which would be transported within a ±0.5% momentum acceptance window (156), CC-BY-3.0.

For facilities with isochronous cyclotrons or synchrocyclotrons, the ELST is typically determined by the time it takes for the magnets in the beam transport line or gantry to respond: to ramp, change and settle (55, 56). As the extraction energy is fixed, a degrader and energy selection system – comprising sections of collimators, magnets and diagnostics – is used to physically attenuate the energy, selecting particles within a small momentum range (i.e. 0.5-1%) to produce the appropriate beam energy (**Figure 5**). Although these degrading devices are mechanically actuated, they can be rapidly positioned as fast as ∼10’s msecs (57–59) and therefore the longer magnet ramping time for beam transport is the primary contribution to the ELST.

Synchrocyclotrons such as the gantry-mounted Mevion S250 and S250i (which offers PBS), and the compact S250-FIT report a ∼50 ms ELST as there is no transport line, rather the beam is modified in the nozzle. The beam energy is specified by moving 18 polycarbonate plastic plates of varying thickness (the thinner plates are positioned upstream) in the beam path, driven by individual motors. The available combination of multiple plates provides a range resolution of 2 mm where switching between plates is optimised for delivery time and does not need to adhere to conventional high-to-low energy sequencing (60). As there is no refocusing or energy selection, this consequently produces large beam spots with increased penumbra due to scattering (61, 62). Therefore beam shaping hardware is necessary for adequate clinical performance, where two opposing sets of leaves – adaptive aperture (AA) – are used to collimate the low dose tails of each spot either dynamically or in static mode (48). The increased spot sizes due to this energy modulation method require AA for penumbra sharpening: this is important particularly for targets at shallow to intermediate depths as scattering in the patient dominates the lateral dose at large radiological depths (63, 64).

### 2.2 Beamline energy acceptance

**Figure 6:**
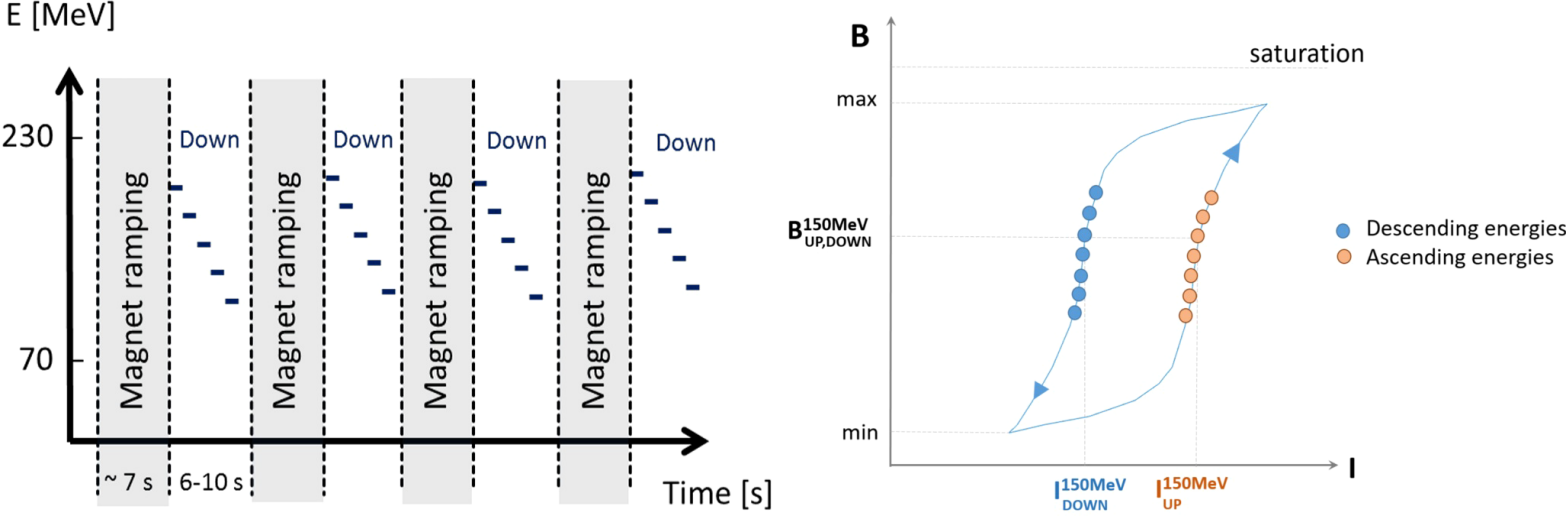
Magnet ramping sequence (shown for rescanning), conventionally from high to low energies (left) (68), CC-BY-3.0. Schematic of a hysteresis loop showing the field current correlation for an electromagnet (right). Stable delivery follows the cycle from maximum to minimum energy when delivering layers (blue circles). Delivering on the ascending side of the loop (orange circles) is performed for energy meandering – a novel delivery strategy (69), CC-BY-4.0.

Beamlines at treatment facilities typically have a momentum acceptance of 0.5-1%. This means that delivery of each subsequent IES (generally varying 2% in energy or ∼5 mm in depth) requires changing the beamline settings: this is the main bottleneck, as the fields produced by each magnet must be changed in order to accept and transport beams of a different energy (65, 66). This is controlled by a set of values corresponding to magnet currents for the nominal fields at each requested beam energy/range (67). The magnets are ramped proportionally to the beam momentum and all involved components (i.e. elements in the ESS) must also be adjusted synchronously. Magnet ramping is driven by current variation with the power supplies but this is limited by hysteresis effects, AC losses and eddy currents (56). Stabilisation of the field (B to within 0.01%) must be retained as small errors can lead to range deviations downstream (21, 25, 55). For example, a variation of just 0.5% in B in the last bending magnet (in a gantry) can cause a 1 mm spot displacement (56). Therefore, to preserve the reproducibility and position of the beam at isocentre, this process of beamline energy regulation can only be performed in small steps, typically moving in one direction (highest to lowest energy) along the hysteresis loop (**Figure 6**). This cycle is established by a first full ramping to initialise the magnet and must be followed to sustain this current to field and energy relation: re-irradiating or changing energies between fields requires re-ramping, still following this sequence (58, 68). One way to move faster between energy steps is by using enhanced magnets and optimising the beamline tuning process, where PSI have reported achieving their fastest ELST of 80 ms (21, 42). However for most cases, ELSTs at the current timescales (see **Table 1**) are a bottleneck delay for PBS treatments: this is a constraint largely imposed by the optics design and could be overcome by increasing the momentum acceptance.

A BDS with a larger energy acceptance can minimise the ELST, leading to faster overall treatments, higher patient throughput and therefore lower costs (70). In addition to better treatment efficiency, a faster BDT does not need to sacrifice or compromise treatment quality (71–73). In contrast, improvements can be achieved with faster delivery, particularly given dose uncertainties due to moving targets which necessitate the application of motion mitigation techniques (74–78). For any given proton therapy system, the time to deliver a single field will vary depending on the tumour volume. Here we define ultra-fast delivery as the ability to deliver a single field within 10 s for most clinical cases. An ideal scenario would be the possibility to deliver the treatment quicker than the timescale of patient motion i.e. within a single breath cycle (*<*6 s), which would negate the need for motion compensation strategies. Nevertheless, even if treatment fields cannot be delivered in such time frames, faster delivery can benefit the implementation of mitigation techniques.

### 2.3 Interplay and motion mitigation

**Figure 7:**
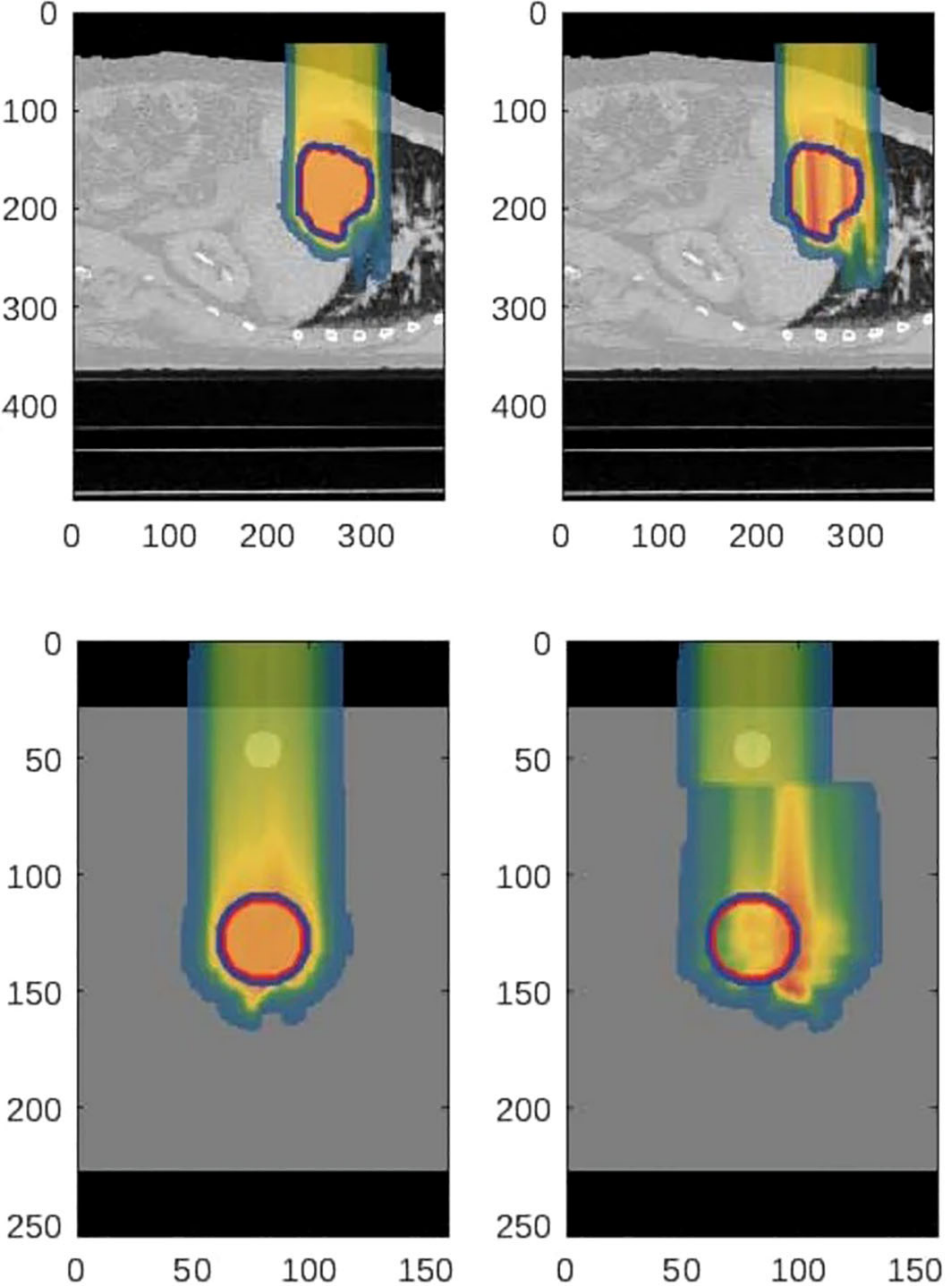
Dose distributions for a stationary target (left) and with motion applied (right), modelled on natural breathing, axes in mm. Resulting inhomogeneities and hot spots are shown when considering a liver case (top) or a 4 cm diameter spherical target (bottom) in a moving phantom, adapted from citation (58), CC-BY-4.0.

Motion has a major impact on the accuracy of any radiotherapy treatment and is a significant challenge particularly in PBT due to the sharp dose gradient, range straggling and dynamic delivery methodologies. Interplay effects (37, 79, 80) are caused by motion between the beam and patient (individually or simultaneously), resulting in a deviation between the planned and delivered radiation to the tumour. Consequently, this produces localised regions of over- or under-dosage which degrade the expected dose distributions and are detrimental to the quality of treatment (**Figure 7**).

Interplay effects can be highly variable as they are dependent on machine capabilities, combined with patient and tumour specific parameters (81). The overall magnitude of motion determines both the suitability of PBT and the extent of dose error caused by these effects: treating moving targets, such as tumours within or nearby mobile organs with PBS is problematic as greater motion will generally result in poorer target coverage (82, 83). For complex cases or sites where there will be large motion (i.e. lung, liver, pancreas etc.) intensity modulated RT or other methods (i.e. double scattered proton therapy) may be recommended instead given fewer associated risks and better clinical outcomes (28, 84, 85). In some cases, the benefit of PBT may still compensate for degradation induced by motion (86).

Intrafractional motion is difficult to control and therefore mitigate, and must be considered on top of interfractional changes (for example temporal and/or anatomical changes such as tumour shrinkage or growth, weight loss etc.) which are commonly experienced during the course of any standard radiotherapy treatment (87). Any differences in geometry can have a significant impact on treatment quality and must be accounted for. Especially for PBT, dose or delivery errors are a greater concern due to the increased sensitivity to inaccuracies and dosimetric precision required. The impact of target motion can be more significant than interplay effects and is shown to increase with time (88). Target drifting is evident even when using immobilisation devices and has a greater impact on planning target margins, but can be minimised by reducing total treatment time (89).

Furthermore, motion within the irradiated areas and in the beam path can affect the delivered dose distribution as this changes the radiologic path length (90). Treatment quality is correlated to the utilisation of the steep dose gradient and therefore influenced by the ability to predict the position of the BP, and deliver the beam to the correct depth (91). Range uncertainties, limitations with *in-vivo* imaging and verification, and inaccuracies with CT conversion factors, all pose as added challenges with motion (92). There are many active respiratory motion management methods which are used by clinics including breath-hold techniques and beam gating or tracking – these are not specifically discussed here, more can found in (93, 94). The effectiveness of these can depend on applicability to the patient, clinical criteria and facility experience, as well as the capability of the equipment and accelerator. Dose inhomogeneities can also be exacerbated if the patient motion synchronises with the beam delivery timing (95). For example, the time structure and dynamic control of pulsed beams with synchrotrons may result in worse treatment quality if the spill corresponds with patient respiratory cycles (i.e. 2–6 s) (96). This may also be a factor with using techniques such as MEE, if the spill length is only able to cover a limited number of IES. Implementing one or a combination of these methods can help compensate for motion however often add complexity to the workflow, requires additional resources, expertise and most significantly, prolongs treatment times. Consequently, improving quality again comes at the cost of treatment efficiency; faster beam delivery could reduce the impact of interplay and allow more effective use of these motion mitigation approaches.

### 2.4 Optimisation and novel delivery schemes

It is clear that having an increased energy acceptance could speed up delivery times, assuming the technical hurdles to realise such a system can be overcome. At present the ideal range is yet to be defined. To fully encompass the 70–230 MeV therapeutic energy range for PBT (i.e. equivalent to 4.8–33 cm water equivalent thickness), a momentum acceptance range of ±30% is required (or -49% to +62% in the energy domain, given a reference energy of 140 MeV). This is dramatically larger than the standard acceptance range and designing a system with this capability has proven challenging (discussed in Section 3). Alternatively, some proposals split up the acceptance range into multiple ‘treatment bands’, delivering a smaller subset of energies such that the BDS only needs to vary if treatments require switching between these bands. It is noted that not all energies across the entire clinical range are consistently used for treatment, rather it is selective based on the treatment indication and patient plan (97, 98). In general, a LEA may reduce the ELST and BDT such that the entire field could be delivered at much faster timescales negating or minimising motion effects, but this has not yet been investigated in detail.

**Figure 8:**
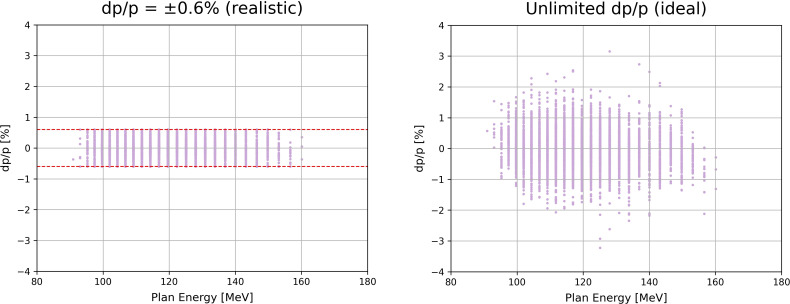
Delivery optimised for a simulated 4DCT plan accounting for breathing motion during tumour tracking for a momentum acceptance range (dp/p) within a realistic acceptance range of ±0.6% (left) and without limitation (right). Adapted from citation (100), CC-BY-3.0, data used to generate image was provided by author Giovanni Fattori.

Implementing optimisation schemes which account for delivery with a LEA is a brand new paradigm: assessing treatment planning and dosimetric impact is only just starting to be explored in the literature but more must be done to understand the potential benefits and effects. This includes examining any benefits with faster ELSTs, novel bidirectional strategies and other variations in beam parameters. A recent study by Wang et al. (99) for a BDS with a -6% to +7% acceptance showed that delivery efficiency could be significantly improved without reducing plan quality and robustness, however there was a sensitivity to spot position uncertainty and concerns about beam shape distortion which was not modelled. Another study by Giovannelli et al. (100) demonstrated that a momentum acceptance range of ±5% was sufficient to cover the variability of large organ motion for a set of lung patients when using rescanning and tumour tracking strategies (**Figure 8**).

The number of energy layers is not typically a variable parameter in the optimisation process by commercial TPS (43, 101). However, several studies have employed energy layer reduction strategies to optimise plans for efficiency, demonstrating negligible difference in plan quality. It was also noted in (102) that IMPT optimisation is highly degenerate: different parameters and plans can yield equivalent dose distributions, and additional variables can offer multiple degrees of freedom with plan optimisation. In this study, the authors were able to decrease the number of proton energies and spots used whilst maintaining the dosimetric criteria. Greater benefits were shown for larger tumours and slower ELSTs; other strategies have also been developed to reduce layers whilst maintaining plan quality (103–106). Ultimately, utilising a faster machine will yield greater efficiency gains than implementing planning optimisation methods, as there is a limit to the achievable reduction in BDT before it comes at the expense of treatment quality (73): the ELST will still remain a barrier to the BDT (107).

Further gains in efficiency can be realised by combining fast delivery with improvements to existing magnet and accelerator technologies, by increasing both scanning speeds and beam current. Particularly with the emergence of methodologies such as FLASH and arc therapy, a LEA BDS may prove essential as an enabling technology given that ELSTs need to be fast enough for rapid energy modulation. For example, for BP (or conformal) FLASH, the whole field likely needs to be delivered in less than 1 s (108), provided that the high dose rate can be sustained for the whole treatment volume, as necessary to elicit the FLASH effect. Transmission or ‘shoot through’ beams are being used instead however are not an optimal solution as they neglect advantages of the BP (109). Passive devices such as ridge filters (110) and 3D-printed range modulators (111–114) are also being explored to produce a conformal depth distribution across the full volume, using a single energy. However, these must be verified for production accuracy and robustness (115) and may require adjustment to the accelerator, BDS and nozzle (e.g. beam monitoring devices) to achieve increased transmission (116). The feasibility of a LEA beam delivery system with a range shifter for BP FLASH delivery was also studied in (117, 118), showing better normal tissue sparing. Nevertheless, there are still many existing challenges and developments needed even with current system capabilities to be able to deliver FLASH dose rates safely and accurately for treatment (119). These are not discussed in this review but more can be found in (120–123).

For proton arc therapy, flexibility in the complex delivery sequencing and ensuring robustness with good treatment efficiency necessitates a fast BDT: ELSTs are also a bottleneck (124–127). Arc therapy is proposed to be delivered either statically by stacking discrete (step-and-shoot) fields – potentially at multiple layers – at each angle; or dynamically (beam stays on during the gantry rotation) with single energy layers per discrete direction (128). As there are still many technical barriers to dynamic delivery, static delivery has been investigated as a feasible pathway towards implementing arc therapy in the clinic. Treatment planning studies performed in (129) showed dosimetric benefits specifically for head and neck (H&N) treatments and delivery times comparable to multi-field optimisation plans, leading to world first patient treatments with static proton arc therapy. In a recent study in (130), the feasibility of proton arc therapy given both delivery modes and the fundamental limitations with a synchrotron accelerator were assessed. The authors demonstrated the possibility for higher quality plans but at the cost of significantly increased treatment times relative to conventional IMPT delivery – even up to 131% longer BDT for a chordoma case – primarily constrained by their 2 s ELST. Additionally, gantry rotation speed is also a limitation (131) and upright patient rotation has been suggested as a method of facilitating proton arc therapy (132, 133). The renewed interest and development into upright chairs and gantry-less systems also offer another parameter space for improvements to treatment, delivery efficiency and the patient experience (134). These opportunities and considerations are discussed further in (120, 135). There are still several challenges to be solved to be able to treat a wide range of sites efficiently with proton arc therapy. Research and development to understand the hardware and software complexities of arc delivery, alongside considerations of safety, workflow, quality assurance, treatment planning optimisation and design have been growing: more is outlined in (136).

### 2.5 Rescanning and bidirectional delivery

Rescanning (repainting) is a technique commonly used to mitigate interplay, where the treatment volume is re-irradiated multiple times to ensure sufficient dosage and to average out motion effects which may cause ‘hot’ or ‘cold’ spots. A minimum number of rescans is required to achieve a more homogenous distribution however, rescanning is usually combined with other active motion management techniques to compensate for motion induced dose conformity losses (93). Different rescanning strategies exist; layered rescanning (LR) is most typically used, where an IES is repeatedly irradiated until the spot objectives are fulfilled (spots are weighted and/or divided per number of rescans) before changing to the next energy layer. In volumetric rescanning (VR), the delivery of rescans is instead ordered by energy layers where each IES is irradiated once before moving to the consecutive layer, scanning across the complete target volume repeatedly. Rescanning can reduce the effects of motion but there is often a trade-off with treatment time. The feasibility and effectiveness is dependent on several factors including key motion parameters (respiration phase, period and amplitude) and technical specifications such as scanning speeds, ELST and beam current (84, 137). Therefore the characteristics and performance of the BDS plays a major role: effective rescanning requires a system capable of precise, fast beam delivery, and quick energy changes (78, 138–140).

Both rescanning methods have been examined in numerous studies demonstrating ranging benefits, dependent on patient requirements and machine characteristics. The time structure of delivery is significant, where deliberate interruptions such as random pauses or variation in the scan path can interfere with correlated motion between the beam and target (94). VR has further potential advantage, offering a greater number of scan paths (90) to stochastically distribute the rescans over the breath cycle, rather than a limited subset as with LR (141). Published results have shown similar plan improvements using both layered and volumetric rescanning (137). A comparative study in liver patients found that LR was more sensitive to the starting phase of the breathing period but for an optimal number of rescans, there was little difference in dose homogeneity (142). For scenarios with more rescans, LR was largely preferred as VR became unviable due to slow ELSTs and extended treatment times. Although volumetric rescanning may have statistical advantages (138), its utility is reliant on BDS capability as ELSTs are prohibitive to both BDT and rescanning efficacy (29, 143).

**Figure 9:**
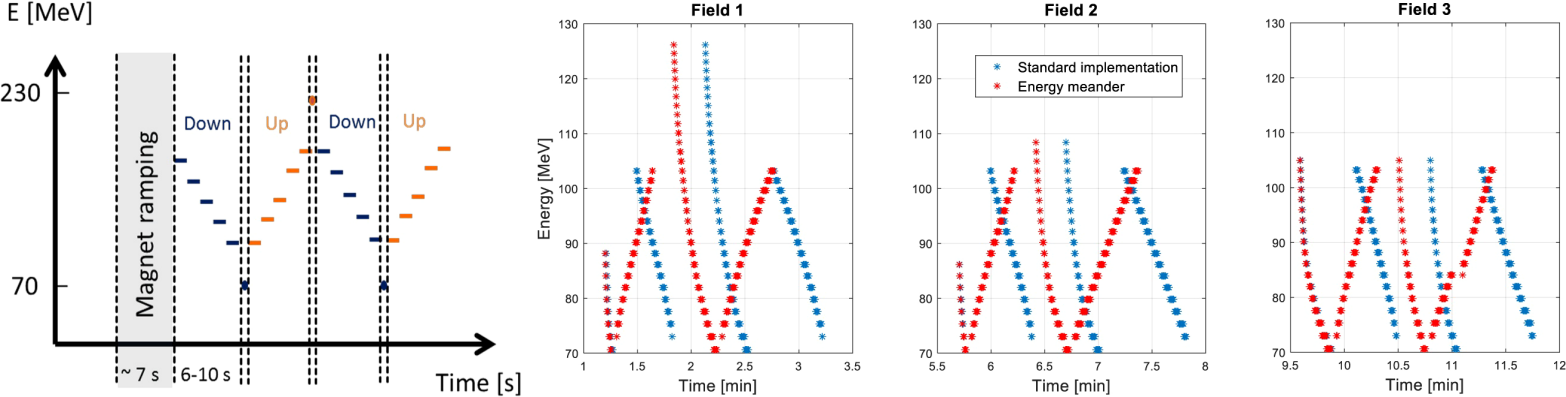
Energy meandering scheme showing ramping profile for both ‘up’ and ‘down’ sides of the hysteresis loop (see also: **Figure 6**) (68), CC-BY-3.0. Energy and time characteristics of a 3 field clinical plan with standard delivery (blue) and energy meandering (red) (69), CC-BY-4.0.

Slow ELSTs are especially challenging for volumetric rescanning, requiring reinitialisation of the magnets in the BDS before each rescan (69). To improve treatment efficiency, operational developments at PSI have shown the possibility of ‘energy meandering’ (**Figure 9**) to allow delivery on both sides of the hysteresis loop to avoid beamline ramping delays (68). This would mean for VR, an additional field could be repainted (in the reverse loop direction) without waiting for another full ramping cycle. Energy meandering has already been implemented for treatments, reporting a reduction of up to 55% in BDT. Although small differences in beam characteristics were measured for equivalent beam energies (of each ramping direction) in this study, QA checks verified outputs were within tolerance. Gamma pass criteria were met showing no dosimetric differences and deviations did not exceed 1-2%, comparable to daily variations. Therefore, precise delivery is possible in both ascending or descending energy directions, enhancing standard ramping schemes restricted by hysteresis effects.

Similarly, ‘Field Regulation’ is a feature implemented by IBA to reduce the ELST in the ascending irradiation direction for volumetric rescanning (144). Hall probes are mounted inside certain groups of magnets to perform real-time measurements providing reference field values (corresponding to the desired range/energy), which are used instead of the magnet current set points. There is some redundancy in these lookup table values as each current can correspond to more than one magnetic field value. The magnets must be ramped again if changing to the opposite direction, thus regulating layer changes by field measurements instead establishes a more direct correlation (145). There are still considerations with measurement reliability and QA procedures before it becomes clinically adopted however, there is incentive for implementation given ELSTs for the ascending energy direction is an order of magnitude greater (from ∼700 ms to 5.5 s, reported for a IBA S2C2 synchrocyclotron) (47).

Energy meandering and field regulation may offer as alternative strategies to speed up delivery in existing systems but there is still a maximum limit to how fast you can regulate or change magnetic fields: these constraints would not be necessary for a LEA system. Ideally, the advantages of a LEA BDS could be fully exploited if combined with an accelerator capable of rapid beam energy variation. A linac and Fixed Field Accelerator (FFA) have repetition rates ranging from 100’s Hz to kHz, meaning pulse-by-pulse changes could achieve ELSTs an order of magnitude faster than currently possible (anything faster than ∼1 ms would defer to the timing resolution of dose monitoring (146) and control system interlocks (147)). The Linac for the Image Guided Hadron Therapy (LIGHT) system was previously under development, using novel high frequency RF accelerating structures for a commercial PBT solution. Construction and testing of the initial prototype was carried out, demonstrating fast energy modulation with repetition rates of 100 Hz (i.e. 10 ms ELST) (148). Several FFAs have been constructed for various applications: a conceptual design study (PAMELA) was proposed for charged particle therapy (CPT)^3^ with a goal of 1 kHz repetition rates to enable ms ELSTs (149). These and other technologies (discussed in Section 3.1) are still under development but may be commercially available in future, as the field evolves to capitalise on the benefits of faster treatment delivery.

The potential enabled by a LEA BDS has not yet been explored in detail and another possibility is longitudinal, bidirectional scanning. With a LEA, there would be minimal difference in changing the direction of depth scanning: delivery would not need to follow the conventional sequential approach of maximum to minimum energy layer. Comparable to using the ascending energy mode in volumetric rescanning, bidirectional delivery could be especially helpful for beam (or tumour) tracking for mobile organs, as well as reduce BDT for future techniques such as proton arc therapy (47). The option to go either direction – whilst at any point during the treatment – also offers additional parameters to optimise for treatment planning. Given this is a new paradigm, very little currently exists in literature relevant to this area: one factor with LEA delivery which may need to be considered is the division of dose across multiple layers. At faster timescales IES delivery may occur effectively simultaneously, in non-discrete layers – an associated concept is explored as oblique raster scanning in (150). For some facilities which use continuous PBS (rather than discrete spot scanning) some fraction of dose outside the prescribed dose may be delivered between spots. This occurs transiting between spots or from a time lag between the system controls to turn off the beam (flap dose) (151), but can be considered negligible in practice (152). However, for the case of a LEA system the intra-layer dose must be evaluated accurately as rapid dose delivery must be able to be controlled and effectively monitored to ensure patient safety.

Evidently, the capability of the BDS determines how fast a treatment can be delivered: minimising the ELST can reduce treatment times and motion induced dose degradation effects, whilst also improving the effectiveness of motion management techniques, and the range of sites PBT could be used to treat. Ultimately, the optimal treatment is one where adequate coverage, conformity and robustness can be achieved, to meet patient requirements. Future delivery techniques are under development and have the possibility to revolutionise the capabilities of PBT (153), but these can only be made possible with advances in beam delivery technologies. The fundamental limitations of existing beam delivery systems can by addressed by increasing the acceptance range, minimising conventional ELST and enabling ultra-fast delivery.

## 3 Technology developments: increasing beamline acceptance to enhance beam delivery

In principle, facility designs aim to fulfil several operational requirements for treatment, where the accelerator and BDS must be able to reliably provide precise (longitudinal and transverse) beam control and sufficient beam intensity with good overall up-time (65). The beam intensity, emittance and momentum spread are important parameters as the machine must be able to efficiently accelerate particles to consistently generate a reproducible and stable beam with a small spread to reduce losses. The BDS design and optics must match and accommodate beams depending on the accelerator and delivery requirements. In the past, the choice of the accelerator was restrictive due to the available magnet and accelerator technology which determined several factors including size, cost, maximum energy, temporal beam structure, delivery characteristics, and achievable treatment quality (154, 155). Significant technological developments have since enabled modernisation to PBS delivery regardless of accelerator type.

A beam with too large an energy spread (or momentum spread) is not ideal for treatment as this determines the modulated dose gradients – the sharpness of the beam – which can impact the uniformity and achievable conformity of the resulting dose distribution (**Figure 4**). When degraders are used to introduce energy losses, it results in particles with a spread of energies. It is useful to design a degrader which reduces scatter and therefore spread, as this increased beam distribution results in lower transmission and beam intensities, particularly at lower energies. A significant proportion of the primary beam is lost at the energy selection system (ESS) which includes dipoles, collimators and an adjustable slit which control the energy spread and emittance: only particles at a dispersive point corresponding to the nominal beam energy are transported (**Figure 5**). A small acceptance in momentum (i.e. ± 0.5%) restricts particles to within a nominal energy window which retains a sharp, steep distal BP fall-off for individual energy layers (156). The sections of beam transport downstream of the ESS may have an equivalent acceptance range for achromaticity: matching this requirement seeks to maximise transmission and also ensures accurate longitudinal positioning (157, 158). Lower transmission through the degrader and ESS can result in more unwanted radiation being produced upstream, which may require greater shielding and therefore costs (21). The output beam intensity can be increased by reducing the occurrence of beam losses at any point within the BDS, as lost particles can also lead to the production of neutrons and excess radiation and damage, or cause quenching of magnetic components (159).

Historically, passive delivery techniques needed a beam with a fixed maximum energy, producing single fields practically instantaneously through range modulating devices, scatterers and absorbers positioned far downstream. Matching beamline properties was advantageous to maximise transmission and made it easier to use a single library of devices (158). Similarly for synchrotrons, matching conditions supported beam stability and transmission, to produce beams with suitable currents and parameters before modification by passive devices (160). Nevertheless, now to deliver PBS with these existing BDS capabilities, the small matched acceptance conditions have become a limiting factor, introducing an ELST each time a new nominal energy is selected and transported for treatment. As long as the beam is defined with the desired characteristics prior to transport through the BDS – which could be designed to be able to accept all particles in range – the momentum acceptance range of the system does not need to be prohibitive. The benefit of a BDS with larger acceptance is not to deliver beams with a large energy spread, which may have a potential impact on treatment quality: rather it enables the delivery of beams of a large range of energies. Therefore, increasing the acceptance range can address the design constraints of existing beamlines and decrease time spent waiting for system adjustment.

Beam transport and delivery systems for PBT have several boundary conditions. The main requirement for the delivered beam is to have an approximately circular spot shape with minimal distortion and be well defined in both position and momentum. In cases where the beamline momentum acceptance is small, with magnets or collimators to ensure correct matching, it can be assumed that this spot is energy-independent in the absence of errors. When considering a large energy acceptance beamline, there are additional constraints also on the accelerator design. In order from most to least important, these are:

1. Trajectories for all momenta converge at the start and end of the beamline (geometric achromaticity).
2. All momenta have the same beam parameters at the end of the beamline (optical achromaticity).
3. Minimal momentum-dependent displacement in the dispersive plane during transport (small excursion).

In accelerator literature, a beamline or insertion is usually regarded as an ‘achromat’ if the beam trajectory is energy-independent at the start and end of the section i.e. the beam dispersion and its derivative are zero. Here, we extend this to make a clear distinction between ‘geometric achromaticity’ – matching the definition previously given – and ‘optical achromaticity’, meaning that the beam shape and size is also energy-independent (i.e. zero chromaticity in the optical functions of the lattice).

Geometric achromaticity is of paramount importance for an LEA BDS, as any residual dispersion at the nozzle would be directly detrimental to treatment quality. Optical achromaticity is also desirable, as this ensures consistency in beam spot shape and size, though perfect optical achromaticity may be impractical over the full range of treatment energies. Although the size of the beam excursions has no impact on the treatment quality, large excursions necessitate large magnet apertures which would increase the cost of a PBT facility and may prevent retrofitting in existing facilities.

### 3.1 Accelerator considerations and prospective magnet technologies

In most accelerators, optical achromaticity is fulfilled sufficiently well by ramping the strength of focussing magnets to match the beam energy, and any residual chromaticity is countered by the addition of sextupole and other higher order magnets. This is not necessarily the case in LEA arcs, which usually do not include magnet ramping. Here, we define a ‘large energy acceptance’ arc as one comprising a geometric achromat over the full range of energies, as this is the minimum requirement for rapid beam delivery, with optical achromaticity as desirable but not a necessary condition.

Many of the design requirements of LEA beamlines are the same as those for a conventional BDS. For example, the horizontal and vertical beam parameters at synchrotron based facilities must be matched at some stage in the BDS, which can be achieved in conventional systems using scattering foils with collimators or matching quadrupoles (161). There is significant additional complexity introduced in a LEA system as particle-matter and particle-magnet interactions are strongly dependent on the beam energy. For example, the focussing strength of a given magnet is inversely proportional to the beam momentum meaning that optical achromaticity is impossible without either rapid ramping magnets or by using energy-dependent trajectories to cancel out the differences in focussing. As such, although the challenges for conventional and LEA beam delivery systems are similar, the solutions are different.

In conventional beam delivery systems, the strength of all magnets must be ramped synchronously with the beam energy to ensure proper beam steering and focussing. This magnet ramping bottleneck is circumvented in LEA arcs by fixing the field of some or all magnets in the beamline, at the expense of energy-dependent particle trajectories and beam dynamics. Although Fixed Field Accelerator (FFA) facilities have been proposed several times for charged particle therapy (149, 162–166), none have yet been realised in the clinic; the same is also true for FFA beam delivery systems.

Future accelerator and beam delivery systems will rely on advanced magnet technologies, for either improved performance or for further size reduction. One innovation being explored for protons is the use of permanent magnet Halbach arrays. A number of recent accelerator test systems have demonstrated the potential for nonlinear fields and field correction techniques (167) and further radiation resiliency tests are underway (168). Whilst much of our focus has been on protons, heavier ions (e.g. helium and carbon) are even more challenging to reach a future scenario of smaller systems and faster treatments, or a vision of single-room facilities. What is clear at present is that the costs and physical requirements for CIBT gantry facilities are higher than for protons. This drives two potential directions: either gantry-less treatments, which is the standard choice at most of the existing CIBT facilities (6), or the use of innovative superconducting magnet gantries. Whether the future will see upright treatments for CIBT is uncertain (169), but partial patient rotation may allow robust angle selection (170) whilst easing gantry requirements.

To first order, the challenge for heavier ions lies in the much higher required magnetic rigidity (over 3 times that of clinical protons) to reach equivalent depths for treatment. This means either the magnetic field strength or facility size is naturally larger than for protons. For example, the first carbon gantry was the Heidelberg Ion Therapy gantry (171) which is normal conducting, 25 m long, with a radius around 6.5 m and weight around 600 t. It is clear that superconducting (SC) technology can help reduce the gantry size: an SC carbon ion gantry made by NIRS/QST and Toshiba (172) is in use clinically, weighing around 300 t, 13 m long and is 5.5 m in radius. This lighter gantry utilises novel 2.7 T SC magnets which are combined function and (relatively) fast ramping (tested up to 0.3 T/s), contained in helium-free cryocoolers to overcome issues arising from the physical movement of SC cryostats. Ongoing work aims to achieve a design reaching a 1 T/m ramp rate (173).

**Figure 10:**
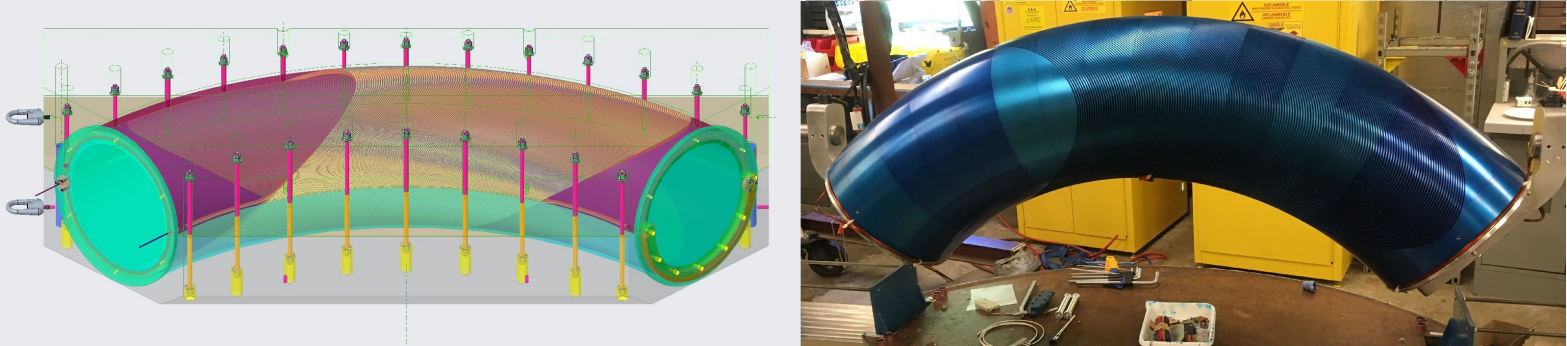
Rendering showing the CCT dipole layers (left) and photo of the fabricated mandrel assembly (right). Images provided with permissions to reproduce by Lucas Brouwer.

In addition to direct-wound SC magnets (cosine-theta type), a possible configuration for SC magnets is Canted-Cosine-Theta (CCT) (174), which can produce pure dipole fields whilst cancelling unwanted solenoidal fields due to its winding geometry, over shorter lengths than conventional cosine-theta magnets (175, 176). Whilst CCTs have not yet been implemented in accelerators or gantries, other single-pass optics studies (177, 178) have presented gantry designs based on curved, alternating gradient (AG)CCT configurations, consisting of focussing-defocusing quadrupole layers inside CCT dipoles (**Figure 10**). As previously shown, Wan et al. report a momentum acceptance of ±12.5%, where an AG-CCT arrangement could achieve the strong focussing required to transport the wide range of rigidities without fast magnet ramping during treatment. The bore radius of the magnet must also have a good field region (min 10×10 cm^2^) to accommodate a suitable treatment field size at the patient isocentre (179). The exact design parameters for the CCT magnet will depend on the optical design of the BDS to determine what higher order field gradients are tolerable.

This new technology also encourages opportunities that are currently beyond current clinical realisation: a LEA BDS as an alternative to a gantry for CIBT or multi-ions could also potentially allow multi-ion transport, remove the need for fast-ramping magnets, and reduce the complex cooling configurations required to enable rotation. The full energy ranges of He-4 and C-12 from 70 MeV/u to 430 MeV/u requires ±46% momentum acceptance, extending to ±69% to also include protons^4^ (181). Achieving this could also facilitate the potential of using helium ions for *in-vivo* imaging (182, 183) alongside C-12 as mixed beams for treatment and online imaging (184).

### 3.2 LEA proposals for charged particle therapy

There are several proposed LEA beamline designs for charged particle therapy, relying on different techniques to achieve achromaticity. The mechanisms used to achieve the large energy acceptance have varied over time with the discovery of new FFA techniques, ranging from linear magnets with a high packing factor to highly nonlinear arcs. The key details of notable proposals are given in **Table 2**. Here we define the acceptance Δ*_p_* of a beamline as

**Table 2:** Proposed large momentum acceptance beamlines for charged particle therapy.

Source	Principle	Rig. Range [Tm] (±%)	KE [MeV]	Geometric	Optical
Keil et al. (2007) 162)	Linear FODO with matching	1.40-2.43 (26.8)	90-250	No	No
Fenning (2011) (185)	Nearly-scaling FFA dispersion suppression	1.23-2.43 (32.8)	70-250	Yes	No
Wan et al. (2015) (177)	AG-CCT dipole/quadrupole (3 settings)	1.4-1.8 (12.5)	90-144	No	No
Brouwer et al. (2019) (186)	Large rectangular bends with variable matching quads	1.23-2.27 (30)	70-220	Yes	Yes^7^
Nesteruk (2019) (59)	Combined function up to sextupole	1.70-2.32 (15.4)	130-230	Yes	No
GaToroid (2020) (187)	Superconducting toroidal magnetic field	1.23-2.43 (32.8)	70-250	Yes	No
Trbojevic (2021) (188)	Edge angles	1.19-2.43 (34.4)	65-250	Yes	No
Dascalu and Sheehy (2021) (189)	Adiabatic transition between arcs and straights	1.23-2.32 (30.7)	70-230	Yes	No
Liao et al. (2024) (190)	AG-CCTs and CF quad/sext magnets	1.69-1.99 (8)	129-172	Yes	No
Steinberg et al. (2024) (191)	Combined function FFA demonstrator beamline	0.1-0.25 (42)	0.5-3.0	Yes	Yes

In cases where multiple species can be delivered, details are given only for proton beams. The final two columns detail whether the arc comprises a geometric and optical achromat.


$${\text{Δ}}_{p}=\pm \frac{p_{max}-p_{min}}{p_{max}+p_{min}},$$

where *p* is the momentum of a particle beam and the acceptance is given by a single number^5^. The rigidity acceptance is the critical parameter when considering a LEA beamline, as there is direct proportionality between the beam rigidity and the necessary magnetic field strength.

The first BDS designed to transport all treatment energies without changing magnet excitation was proposed by Keil, Sessler, and Trbojevic (162) in 2007. The ‘KST’ gantry, designed as part of a wider facility for CPT, comprises many repeating cells of combined function dipole/quadrupole magnets. An insertion of two quadrupoles and drifts is used to connect the beam transfer line to the gantry, matching a circular beam spot to the rotating gantry. The gantry is made up of three quarter-circles with a radius of 5.27 m, giving an overall footprint of approximately 16.5×8.5 m^2^. The magnet packing factor for the gantry is not given but the drifts appear much shorter than the magnets. As the KST design uses one periodic cell with linear optics, only the reference energy can be properly matched: as details for other energies are not given, presumably they are mismatched both geometrically and optically. Given these caveats, the KST lattice design however still demonstrates the potential of a LEA BDS and establishes a good benchmark for further work.

Fenning and Machida (185) propose an alternate design methodology, using ‘scaling’ FFA optics (where the phase advance as a function of energy is fixed using nonlinear magnetic fields) to create a dispersion suppressor that brings all trajectories together, to first order. This is then optimised to bring together all the closed-orbit positions at the end of the arc, with both zero dispersion and its derivative. In this work, several alternate gantry designs are presented with magnets assumed to be similar to those in the PAMELA study (149). It is found in all cases that sufficient dispersion suppression can be achieved, however the beta functions are severely mismatched leading to an energy-dependent spot size. It is suggested that this beam size variation can be countered as part of the scanning system, but detailed studies have not been performed.

Another design strategy is presented by Dascalu and Sheehy^6^ (189), using connected ‘adiabatic transitions’ where the bending strengths of combined-function magnets are gradually increased/decreased over several magnets along the arc. This slow variation should ensure that the beta functions are not perturbed but the dispersion should smoothly come back to zero. Using adiabatic transitions leads to a large footprint – approximately the same as the KST lattice – and requires a very high packing factor, with 58 cells in total required to ensure good matching. Despite the large number of magnets, the results indicate that the residual dispersion at the end of the arc is almost as much as in the main part of the arc itself, though the orbits converge well. However, with such a large number of magnets in close proximity, it is not clear that an adiabatic transition would be suitable for a PBT facility.

Trbojevic et al. (188) provides another technique to achieve geometric achromaticity whilst maintaining linear accelerator optics, making use of magnet edge angles to bring all trajectories back together. With this method, it is not possible to produce an optical achromat, as the small path length variations do not compensate for the differences in focussing from the quadrupoles. In addition, the lattice packing factor is just as high as for an adiabatic transition. The authors suggest that permanent magnet ‘Halbach’ arrays can be used to provide the high field gradients necessary to ensure that beam excursions remain small, producing gradients in excess of 150 T/m, though this would preclude reductions to the facility footprint by adopting SC magnet technologies. Though this arc design technique shows some promise, detailed studies on the impacts of realistic magnet fringe fields and errors have not been performed, making it difficult to assess the feasibility of such a beamline under realistic conditions.

A 2019 design study by Brouwer, Huggins, and Wan (186) achieves a 70–220 MeV proton kinetic energy range using fixed-field Nb-Ti superconducting magnets up to 3.5 T, designed with racetrack coils. A fixed field SC dipole bends the full energy beam into the gantry, which then passes through a gantry-mounted energy degrader. A geometrically achromatic bending section consists of two straight dipole magnets totalling a 155 degree bend, achieving achromaticity in a similar manner to the end regions of a racetrack microtron. For optical achromaticity, three fast-ramping resistive quadrupoles are placed symmetrically on either side of the dipoles to provide optical matching. The strength of each of these three magnets must be independently varied to ensure consistent beam spot shape and size, which may become a bottleneck for energy layer switching. The authors estimate that the design could bring the ELST down to ∼100 ms or less, and that the design is a few metres smaller than competing options.

It is possible to devise other approaches for beam delivery which do not resemble a conventional BDS. One particular design of note is the GaToroid study from CERN (187), which proposes to use a large SC toroid (similar to the ATLAS detector magnet), with the patient couch located at the centre of the toroid. The proton beam is directed into the field of the toroid, where its trajectory curves in the magnetic field towards isocentre, allowing for a wide range of irradiation field angles without needing to rotate the magnet structure or patient. The design requires at least one fast switching X/Y steering ‘vector magnet’ dipole to direct each beam energy onto the necessary trajectory in the toroidal field. It is reasonable to expect from first principles that the tolerance requirements on the rapid switching dipole would be very challenging. The scale of the required vacuum and cryocooling systems to cover all beam angles around a patient may also be prohibitive.

The gantry design by Nesteruk^8^ et al. (59) comprises two combined function dipole-quadrupole-sextupole magnets with one combined function quadrupole-sextupole magnet in between, where each combined function magnet uses a pair of SC racetrack coils. The gantry has a momentum acceptance of ±15%, though the nonlinear fields result in some beam distortion. The gantry is combined with an ultra-fast degrader mounted in the gantry and a 2D lateral scanning system. The analysis presented in the paper demonstrates that a range of ±30% in energy acceptance is expected to enable 70% of treatments to be delivered with a single magnet setting. There is no dedicated energy selection system resulting in a higher energy spread of each pencil beam and the potential influence on beam quality is not discussed. Although the increase in energy spread is suggested to have inconsequential impact on the distal fall-off and avoids dose gradients which are too sharp (54), spot distortions due to increased energy spread will require careful assessment. These distortions are relevant for lateral scanning at large scanning angles, in particular for low beam energies, where the energy spread after passing the degrader is greater than ±1-2%. Mitigation strategies, such as accurate spot-position-dependent beam modelling for treatment planning and adding an adjustable field specific aperture at the end of the nozzle, might be necessary. The gantry design followed an iterative process using different codes for high-order beam transport calculations and particle tracking in the magnetic fields, as reported in (59, 192). Though this design reached a phase ready for magnet fabrication, further work towards this BDS has not taken place.

Liao et al. (190) propose a similar solution, offering a ±10% momentum acceptance which incorporates a movable energy slit in the middle of the achromatic bending section. The slit, together with variable-size collimators in the degrader system, allows control of the momentum spread and beam size; their proposed delivery method is discussed later in Section 4.1. In particular, this solution mitigates spot distortions due to dispersion effects. A disadvantage of this energy switching method is the time required to move the slit and adjust the collimator size, contributing to the total beam delivery time.

At the University of Melbourne, the Technology for Ultra Rapid Beam Operation (TURBO) project^9^ has been proposed to de-risk techniques required to realise a LEA arc via a scaled-down technology demonstrator beamline. The initial design by Steinberg et al. (191) proceeds along similar lines to Fenning’s earlier work, beginning with a scaling FFA lattice before using a multi-objective genetic algorithm to reduce the residual dispersion whilst also maintaining an approximately constant beam size. By employing a multi-objective routine, this methodology optimises ancillary variables, allowing a reduction in the maximum excursion (i.e. a smaller beam pipe and magnet aperture) without degrading the beam quality. However, this beamline requires highly nonlinear optics and optimisation of the multiple components of several magnets up to decapole order, leading to some distortion of the delivered beam spot. Further work aims to improve this result by imposing boundary conditions that ensure geometric achromaticity, and to scale up the TURBO design to clinical energies.

Given these LEA arc designs, it is apparent that there are two main routes to providing the additional degrees of freedom necessary for a large energy acceptance: having a large number of magnets, or increasing the nonlinearity of the allowed magnetic fields. No design concepts have yet been realised however several proposals are being further developed, in addition to innovation in magnet technologies. A LEA system offers improvements surpassing existing technology, enabling a wide range of possibilities for treatment (as discussed in Section 2). The extent of these capabilities will be determined by the characteristics and feasibility of the BDS design.

## 4 Considerations for clinical implementation

Whilst each LEA proposal aims to increase the energy acceptance using various design techniques and technologies, it is also important to consider the different aspects which may need to either be addressed in conceptual stage, reactively in the clinic, or in reality be inconsequential and thus manageable with standard operation. Nevertheless, a LEA system will offer capabilities to deliver novel PBT treatments which have not yet been explored by currently existing systems. In practice, this may result in beam properties different to what is normally ‘standard’ for a PBT facility where some flexibility may be expected with ideal beam parameters and standard machine operation (193). Although clinics experience day-to-day variations with accelerator operation and beam performance, it is important that this can still be accommodated with a LEA system whilst maintaining high performance and beam quality within the recommended clinical tolerances.

An ideal LEA BDS design would be able to be retrofitted universally, without dependence on vendor specific components or complex adjustment to the existing system it joins. However, there may be unavoidable uncertainties and predicted variability with beam parameters, compared to conventional systems. We examine some aspects fundamental to clinical performance such as beam size and shape, discuss how these could be managed in practice, and the relevant considerations for implementation.

### 4.1 Beam properties

**Figure 11:**
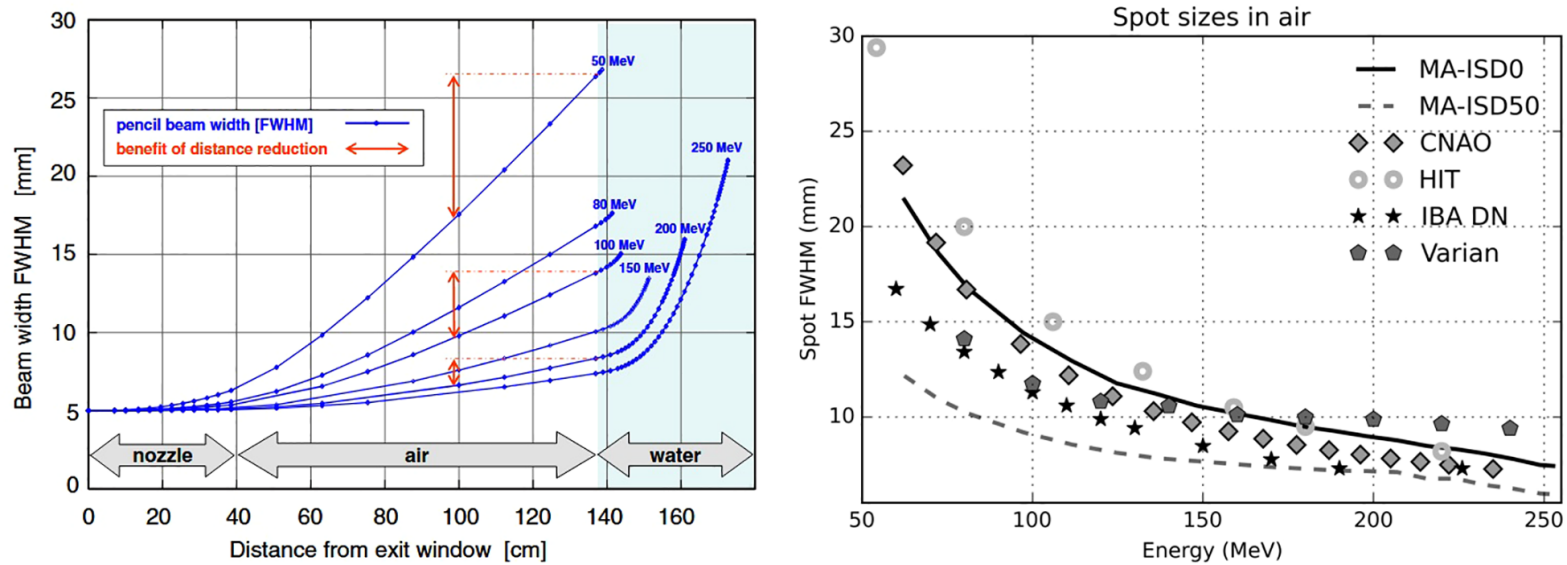
Calculated increases in beam size using the same nozzle geometry for various proton energies measured downstream of the nozzle (left), also showing the effect of reducing the drift distance in air by 40 cm (red) (203), CC-BY-2.0. Measured spot sizes (FWHM) at isocentre in air, at different PBT facilities (right) (196), permissions granted by Rightslink (John Wiley and Sons).

A challenge for most LEA designs (Section 3.2) is the beam distortion caused by nonlinear and higher order effects due to the magnetic field requirements, resulting in non-circular, asymmetric beam spots. The distortion may be significant and have an energy dependence, as spot size and shape vary as a function of gantry angle (194, 195), and energy (196). This may also cause further complication due to amplification downstream at large transverse scanning angles (99). In addition to field effects from the various magnets through the transport line, the beam can also be altered during extraction (197), by other components in the BDS such as beam instrumentation and vacuum chamber windows (198), the nozzle (configuration or type e.g. universal or dedicated) (199), the presence of patient specific devices, collimators (200), range shifters (201) or boluses (202), and depending on other downstream factors such as the air gap and distance to isocentre (196, 203, 204) (**Figure 11**).

Although the beam properties are defined by the BDS optics, the contributions from external factors are likely to have a non-trivial impact on the beam spot. For example, exposure to air and scattering within the nozzle can ‘wash’ out any shape irregularities towards a Gaussian distribution (205) where beam spot distortion may become insignificant when measured at isocentre. However, the extent will vary depending on characteristics such as the initial beam energy, size and shape. The beam delivery system may also need further tuning to achieve ideal performance at isocentre: to produce a symmetric and consistent beam across all energies (196).

#### 4.1.1 Spot variation

The beam size changes with different energies and spots with a small penumbra can lead to a reduced integral dose (206). This is challenging at lower energies as small pencil beams are particularly prone to multiple coulomb scattering (207) which contribute to the low dose ‘tail’ regions, and can dominate the lateral dose distribution (208, 209). This can cause beam broadening and penumbra growth (210) where larger spot sizes can result in reduced conformity (203, 211) and lower treatment quality (212, 213). This increased scatter and beam size is particularly consequential for shallower targets (**Figure 11**) as the increased lateral penumbra and dose fall-off may reduce tissue sparing at low energies (64). At higher energies, multiple coulomb scattering is less prevalent and nuclear interactions dominate the spot size: an initial small beam with a sharp penumbra and fall-off can minimise the spread which occurs within the patient itself (214). However, this can also result in dose inhomogeneities (215) (hot or cold spots) and is more sensitive to motion (216).

Penumbra growth due to interactions within the patient may also be reduced with collimation (211, 217, 218) and patient specific apertures (200, 219). As multileaf collimators are common in conventional RT, collimators were previously explored for PBT (214, 220); also with passive (wobbling) systems in the earlier days of PBS (221). Improvements in conformity or tissue sparing were reported particularly for low energy beams (222), fixed beamlines (63) and irradiation of cranial (204, 223, 224) and H&N tumours (225). The use of any beam modification devices must also consider the consequences of additional contaminant scatter (226, 227), activation, secondary particle production; principally, neutrons (36, 62, 228) and increased surface, physical, and out-of-field dose (229, 230). A dynamic collimator system consisting of two pairs of orthogonal nickel blades (231, 232) is being developed for PBT, and Mevion offers its adaptive aperture technology, a mini-MLC with a small air gap (48, 60). Both of these systems offer energy layer specific collimation however the sequencing and translation of these devices also introduces added time penalties (233, 234). A concept using a thin metal block with a cylindrical aperture attached to a robotic arm has also been proposed to trim individual proton beamlets (235). The Mevion S250-FIT utilises range shifters for fast energy changes (see Section 2.1) with AA but an additional 5–10 s for delivery of each field has been reported (236). A BDS which implements any auxiliary devices requires that these supplementary components operate in synchronicity, sufficiently fast and with positional accuracy: presently for PBT, interventional methods of beam shaping by dynamic collimation is disadvantageous to the overall beam delivery time.

Similarly with beam size, spot positions can also vary with energy. As described in Section 2.5, the control system uses a lookup table of parameters which correspond to accelerator, beamline and scanning magnet settings to ensure accurate delivery of each spot and maintain beam positioning at isocentre. A LEA system which does not need to rely successive magnetic field changes for each IES on may offer better stability in this respect^10^ however will need to manage any additional dispersive effects (237). A comprehensive QA program will be critical to ensure spot parameters remain within the tolerances recommended by the AAPM TG-224 (±10% and ±1 mm, respectively) (238).

### 4.2 Delivery strategies

Although beam control and spot variability still poses as a challenge for LEA delivery, the possibility of rapid interchange between beams with variable parameters and different planning strategies which capitalise on these advantages, may offer advantages for treatment.

**Figure 12:**
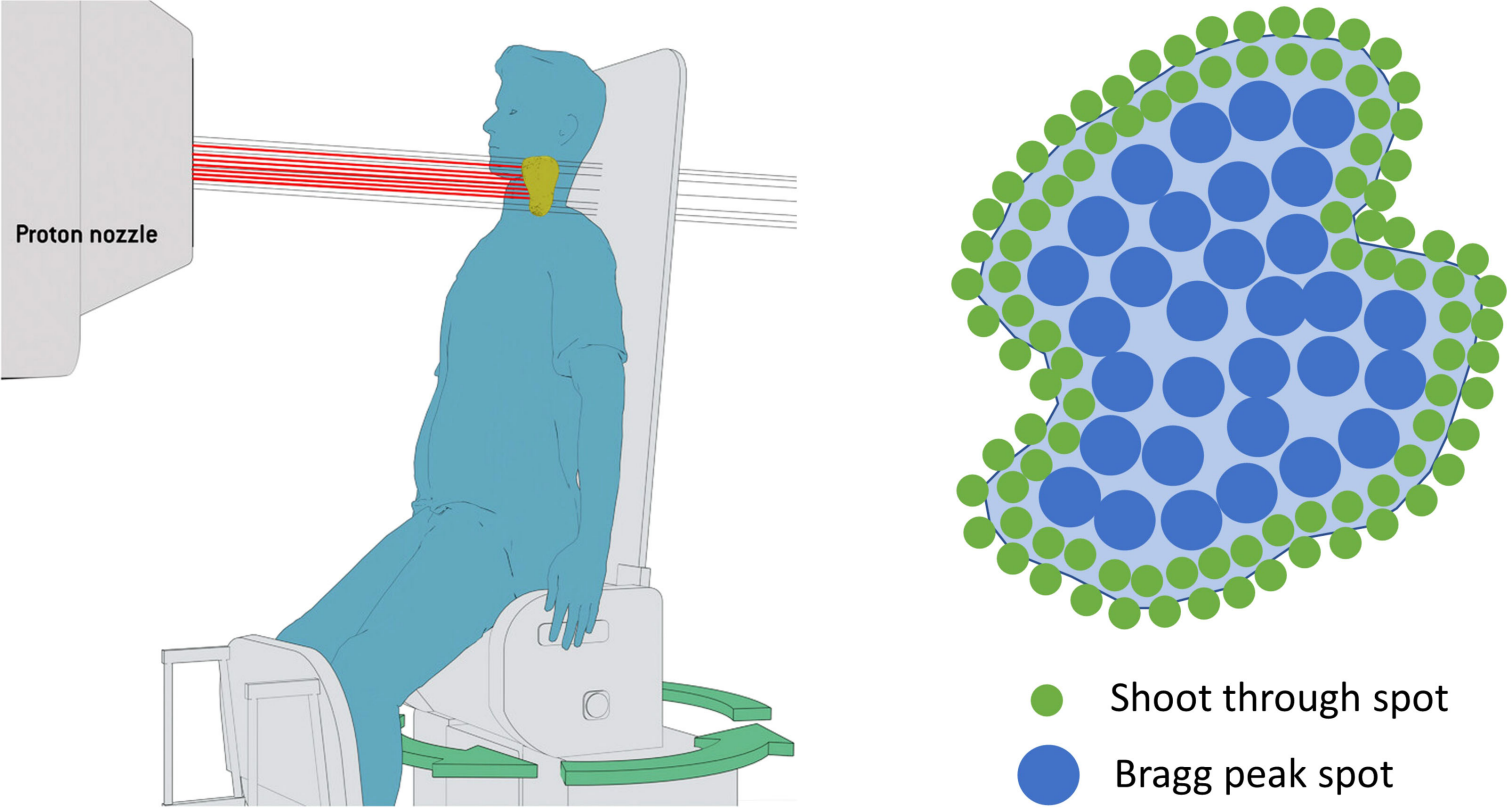
Upright static arc delivery with TBs (left) (234), CC BY-NC 4.0. Illustration showing the arrangement of transmission (‘shoot through’) beams to irradiate small spots at the target boundary with larger, conventional BP spots for the remainder of the volume (right) (240), CC-BY-4.0.

To circumvent energy dependence and spot variation with their LEA system, Wang et al. (239) proposed delivering mixed spot sizes for prostate cases by using adjustable collimators to produce fields with large spots centrally, surrounded by small spots at the periphery (**Figure 12**). A set of collimators and an energy slit upstream of the nozzle restricts the beam to ±0.1-0.5% to produce a small spot and can be opened to return to the ‘natural’ larger momentum spread, transporting larger spots. Small spots are used for the two outermost contours of the target boundary, for each energy layer, and for the entire last energy layer to obtain a sharper distal fall-off. The collimation addresses distortion effects due to dispersion at large scanning angles however adjusting the slit and collimator size compromises the delivery speed which is a disadvantage of this mixed-size method. In practice, trimming the distal dose fall-off may also be counterproductive, as dose gradients which are too sharp increase the sensitivity to machine range errors and can result in hot spots or non-uniformities (54). Spot size has a different impact depending on disease site and there are several technical trade-offs with quality, speed and robustness (198).

Alternatively, the use of transmission beams (TB) have been explored as an approach to instead capitalise on the steep distal fall-off, by improving the lateral penumbra and tissue sparing. Van Marlen et al. (241) initially demonstrated the applicability of multi-field TB plans (for FLASH delivery), where Kong et al. (242) showed the potential for H&N treatments by combining IMPT fields with 244 MeV TBs. Penfold et al. (240) examined the use of a TB (at max 226 MeV) in a single arc to shape the target boundary with IMPT fields for the interior volume. Engwall et al. (234) proposed delivering static arcs with TB for upright treatments (**Figure 12**), reporting high quality plans and improved efficiency over delivery slowed by collimators or ELSTs. Hytönen et al. (243) demonstrated that hybrid IMPT and TB fields could significantly reduce BDT without compromising target coverage for breast and H&N treatments. Similarly, Maradia et al. (236) proposed combined fields with an optimised scanning routine to further reduce BDT.

Additional considerations with radiation protection and beam angle selection for TBs are needed, as the proton range may not exceed the patient. In general, the use of TBs for PBT may add potential improvements to organ at risk sparing and with a LEA beam delivery system, could be delivered instantaneously alongside conventional BP layers.

### 4.3 Energy modulation

Given existing technologies, the most practical method to rapidly change beam energies is through the use of an energy degrader. The design of the degrader determines many beam properties, including: choice of material (lower Z generally results in less scattering and energy spread), geometry (i.e. thickness and shape: wedge, wheel or blocks) (244), and location in the beamline. It must be positioned upstream or configured such that there is sufficient shielding and minimal exposure to the patient, and also be robust enough to move quickly with low uncertainty of positioning error. As the degrader increases the angular divergence, energy spread, and emittance, typically an ESS or multiple downstream collimators are required to restrict the beam to a nominal, narrow distribution. However, this results in losses to particle intensity which is significant (*>*99%) at low energies (157). The absence of an ESS for a LEA system – redundant in this case given restrictive magnet ramping speeds – allows a much higher overall transmission however will also transport particles outside of conventional acceptance limits. This requires the design of the beamline optics and components configured for an optimal balance between transmission, emittance and energy spread^11^. Slits or collimators may be necessary to ensure appropriate beam selection and shaping, but also introduce further practical and physics complexities. Another approach for energy modulation is to employ multiple wedges with different lengths and materials, grouping energy ranges into multiple bands to improve beam properties and optimise delivery efficiency (190).

A narrow momentum acceptance band can be considered an additional layer of safety that prevents an incorrect energy beam from reaching the patient. In the unlikely error scenario where the actual beam energy does not match the settings of the beamline magnets and other interlocks fail, the beam would be transported with significant losses. For a sufficiently large discrepancy between the desired and actual energy – beyond the beamline energy acceptance – the beam will be completely lost in the beamline before reaching the patient. For LEA beamlines, this feature will no longer be present: a robust verification and interlock system must ensure that only the correct beams are transported through the BDS. There are several redundant interlocks in the treatment control system and additional independent methods which can be added to verify that the energy is set correctly. For instance, in the case of a wedge based energy degrader, the degrader control system will receive signals from encoders to verify the position of the wedges. Two independent sets of encoders can be used to provide redundancy. Another possibility is an online check of the beam position, by verifying the beam position in the dispersive region of the large momentum acceptance beamline using a non-destructive beam position monitor. In addition, an efficient way the PBS system could be utilised for verification is to deliver a short beam pulse to a beam stop within the nozzle, at the beginning of each layer, scanned at the maximum deflection angle outside the irradiation field. At such a large angle, this would be sensitive enough to detect a discrepancy between the requested and actual beam energy; an additional spot delivering a small dose to the beam stop also shouldn’t impact the rapid dose delivery capability of the BDS.

The control system must be capable of maintaining effective beamline operation and fast synchronism with all ancillary components (degrader, slits, scanning magnets etc.). Ultra-fast energy switching may be on the order of 10 ms: the system, electronics and interlocks must be able to respond instantaneously, interrupting the beam if there are any detected mismatches with beam parameters. A study performed at PSI investigated the feasibility of rapid and continuous energy modulation, significantly decreasing their ELST by 45% to 27 ms (for small energy changes) (67). The authors developed a strategy to exploit the full beamline momentum acceptance (typical few percent) by parametrising energy bands for range compensation, creating new beamline settings to allow continuous energy regulation. They were able to preserve clinical beam quality but suggest further development to translate experimental performance to clinical settings e.g. calibration of the scanning magnets with the new band settings and optimising the degrader motion and latency can further minimise dead time.

Therefore, there are numerous sources of beam variation in a LEA beam delivery system: additional to nonlinear field effects, the nozzle and auxiliary devices can have a non-trivial impact on the output beam characteristics. This could result in distinct and enduring beam characteristics, where inconsistencies in spot size or shape can lead to dose errors and deviation from planned dose calculations. This requires further study where beam irregularities need to be realistically implemented in the TPS, rather than standard beam models which typically assume a circular spot (199), or a Gaussian distribution in phase space (128). It is possible that some of these features will have a significant or an inconsequential effect on treatment: in practice this may mean any departure from standard operation could actually be negligible as long as variations are clinically tolerated. Given the range of possible errors due to the complex coordination of different beamline elements, rapid delivery requires that all system components cooperate and communicate together quickly and reliably. A large energy acceptance BDS must be designed to operate safely and have the ability to deliver beams which are reproducible, accurate and effective for all clinical treatments.

## 5 Summary

The role of PBT as a primary modality of cancer treatment continues to mature, as improvements in technology and costs drive the rapid expansion of facilities worldwide. The dosimetric and therapeutic benefits are well established, however even if parity with conventional radiotherapy is achieved in terms of accessibility and costs, several limitations still need to be addressed to fully exploit the advantages of PBT. As the future evolves towards emerging methodologies – arc, upright, mixed beams or new particle types etc. – improvements in the technological capabilities of existing beam delivery systems still need to be overcome: not being able to deliver a treatment sufficiently fast is a fundamental constraint, and a key obstacle to enabling superior and advanced treatments.

Increasing the energy acceptance range of conventional beamlines enables rapid energy modulation, ultra-fast treatments, and a multitude of possibilities including volumetric rescanning, bidirectional delivery, proton arc therapy, and BP FLASH. A large energy acceptance BDS could significantly reduce treatment times and costs, whilst enhancing the treatment quality and utility of PBT. Ultra-fast delivery can immediately benefit current treatments by increasing delivery efficiency, throughput and enabling more effective use of modern motion management techniques. Further gains can also be achieved with novel delivery approaches and planning optimisation strategies, however the greatest advantages can only be realised with improvements to the BDS.

Several PBT beamline designs with an increased momentum acceptance range have been proposed and are under development, however none have yet been constructed. These will require further innovation in magnet technology, degrader and collimation systems, and the integration of these components in novel conditions. Efforts in these areas also contribute towards addressing the underlying hurdle of affordability, as developments in the BDS can offer further improvements to the costs and compactness of PBT facilities.

Many considerations also still remain with beam quality, clinical feasibility and performance. These warrant further study to model and evaluate the potential impact on treatment quality, and other potential challenges which may arise: it is crucial this is also investigated in practice through experimental demonstration. We have examined the existing literature to provide critical discussion, identifying opportunities and challenges for clinical implementation. A LEA beam delivery system offers improved capabilities for PBT with ultra-fast beam delivery and beyond, enabling many exciting possibilities for current patient needs and future facilities. 

## Footnotes

1 This extends to carbon ion facilities which are less prevalent where the increased beam rigidity requires much larger infrastructure to accelerate and deliver heavier ions, in addition to complexities with radiobiology.

2 In this paper we refer to the BDS as comprising all the components after the accelerator complex in the bunker, downstream to the nozzle in the treatment room.

3 CPT is used in this paper synonymously with hadron therapy, referring to particle beams of protons or heavier

4 Provided the accelerator can produce suitable particle types at a high enough energy, proton CT requires a maximum energy of 330 MeV (180) and would also be covered by this acceptance.

5 Note that for accelerators where only one charge state is transported, the momentum and rigidity acceptances are identical.

6 Note that Sheehy contributed to this review.

7 Optical achromaticity is achieved using fast ramping quadrupole magnets rather than with a ‘fixed field’ solution.

8 Note that Nesteruk also contributed to this review.

9 The authors also contributed to this review.

10 also for gantry-less systems where there are no gantry dependence errors such as beam deviations or offsets caused by mechanical strain, optics mismatches, or asymmetries at the gantry coupling point (66).

11 An advantage of a LEA BDS is the ability to deliver beams across a large range of energies, rather than beams with a large energy spread.
